# Genomic analyses point to a low evolutionary potential of prospective source populations for assisted migration in a forest herb

**DOI:** 10.1111/eva.13485

**Published:** 2022-10-02

**Authors:** Frederik Van Daele, Olivier Honnay, Hanne De Kort

**Affiliations:** ^1^ Department of Biology, Plant Conservation and Population Biology KU Leuven Leuven Belgium

**Keywords:** assisted migration, climate change, forest herb, genetic offset, landscape genomics, local adaptation

## Abstract

Climate change is increasingly impacting temperate forest ecosystems and many forest herbs might be unable to track the changing climate due to dispersal limitation. Forest herbs with a low adaptive capacity may therefore benefit from conservation strategies that mitigate dispersal limitation and evolutionary constraints, such as assisted migration. However, assisted migration strategies rarely consider evolutionary constraints of potential source populations that may jeopardize their success. In cases where climate adaptation is overshadowed by competing evolutionary processes, assisted migration is unlikely to support adaptation to future climates. Using a combination of population and landscape genomic analyses, we disentangled local adaptation drivers and quantified the adaptability and vulnerability to climate change of the self‐incompatible deciduous forest herb *Primula elatior*. Southern populations displayed a sharp genetic turnover and a considerable amount of local adaptation under diversifying selection was discovered. However, most of the outlier loci could not be linked to climate variables (71%) and were likely related to other local adaptation drivers, such as photoperiodism. Furthermore, specific adaptations to climate extremes, such as drought stress, could not be detected. This is in line with the typical occurrence of forest herbs in buffered climatic conditions, which can be expected to reduce selection pressures imposed by climate. Finally, populations in the south of the distribution area had increased sensitivity to climate change due to a reduced adaptive capacity and a moderate genetic offset, while central European populations were sensitive due to a high genetic offset. We conclude that assisted migration from southern source populations could bear significant risk due to nonclimatic maladaptation and a low adaptive capacity. Regional admixture and restoration of ecological connectivity to increase the adaptive capacity, and assisted range expansion to suitable habitat in the north might be more appropriate mitigation strategies.

## 1 INTRODUCTION

Ongoing climate change will likely have increasingly severe effects on biological diversity (Butchart et al., 2010). Global surface temperatures increased by 1°C compared to preindustrial levels in the last decade (2010–2019), and land temperatures in Europe have increased even faster by on average 1.8°C (European Environment Agency, 2021). Due to these rapidly increasing temperatures together with increasing drought frequencies (Grillakis, 2019), more than half of the plant species in Europe are expected to become vulnerable or threatened by 2080 (Thuiller et al., 2005). Specifically, rapid climate‐change induced shifts of their potential distribution will exceed their adaptive capacity or their ability to migrate to newly available habitat (Kubisch et al., 2013). The strong fragmentation of forest habitats on the European continent makes forest herbs particularly susceptible to climate change due to local genetic erosion and loss of adaptive potential, and due to their limited dispersal capacity (Dullinger et al., 2015; Naaf et al., 2021; Svenning et al., 2008; Van Daele et al., 2021). Assessment of their adaptive capacity is therefore required to predict species‐specific vulnerabilities to climate change, and to devise mitigation strategies against climate change‐induced local extinctions across their range (Bussotti et al., 2015; Razgour et al., 2019).

When plant populations experience increasing environmental stresses due to climate change and are unable to adapt or migrate, assisted migration could prevent their (local) extinction (Hoegh‐Guldberg et al., 2008). Plant populations can be relocated through assisted population migration within their range (assisted gene flow) or outside the range (assisted range expansion) where the climate has become suitable. This requires the identification of suitable source populations and target sites. Source populations should have beneficial adaptive traits and sufficient genetic variation allowing future evolutionary responses (Aitken & Whitlock, 2013). Target sites for assisted gene flow are populations with a high sensitivity to climate change, that is, low adaptive capacity and/or a degree of maladaptation to the future climate (Capblancq et al., 2020; Holderegger et al., 2006; Rellstab et al., 2021). Furthermore, in case of assisted gene flow, careful consideration of the genetic relatedness between source and target populations is required to prevent potential outbreeding effects (Vandepitte et al., 2010), detect fitness trade‐offs (Ågren et al., 2013), and avoid disruption of local adaptation to other environmental factors. Specifically, the introduction of genotypes adapted to environmental conditions varying substantially from those at the introduction sites increases the probability of maladaptation and can imperil evolutionary and migration potential (Wadgymar & Weis, 2017). As a result, such evolutionary constraints may impact the success of assisted migration aiming to facilitate adaptation to warming climates.

Local adaptation is shaped by divergent selection pressure, gene flow and demographic processes, which together drive phenotypic differentiation (Orsini et al., 2013; Savolainen et al., 2013). Local adaptation along geographical or environmental gradients often results in the gradual turnover of allele frequencies or phenotypes. In randomly mating species with large population sizes such clines are generally manifested gradually, whereas species with limited dispersal may be featured by a strong genetic turnover in specific regions of the gradient (Savolainen et al., 2013). The duality of the role of gene flow in local adaptation is that it can either increase genetic diversity and therefore increase the adaptive potential, or disrupt local adaptation by introducing maladaptive alleles (Akerman & Bürger, 2014; López‐Goldar & Agrawal, 2021). When gene flow has been historically low, local adaptation may be more pronounced but genetic diversity could be eroded through genetic drift. Furthermore, there is the danger of outbreeding depression when individuals from locally adapted populations are introduced in more northern populations with the aim to complement them with genetic variants preadapted to warmer climates (Frankham et al., 2011). Forest herbs have been shown to frequently manifest distinct fine‐grained signatures of local adaptation (De Kort et al., 2020; Garrido et al., 2012; Herrera et al., 2017), which may cause strong maladaptation to environmental factors other than climate, following assisted migration. Assisted migration of forest herbs, typically characterized by limited dispersal capacity and gene flow, thus requires careful population genomic analyses to pinpoint suitable source populations, but more sophisticated analyses are required to disentangle climate adaptation from other local adaptations and to evaluate sensitivity to climate change (Aitken & Whitlock, 2013; Vanhove et al., 2021).

Local adaptation to climatic drivers is species dependent (De Frenne et al., 2011), can occur at multiple scales (Csilléry et al., 2014; Pluess et al., 2016; Rellstab et al., 2017), and multiple adaptations in plant species can be related to distinct climatic drivers (Franks et al., 2014; Leroy et al., 2020; Mahony et al., 2020). However, the cost of acclimation to drought and temperature stress can result in fitness and metabolism trade‐offs (Reich et al., 2003; Thiel et al., 2014; Vanwallendael et al., 2019). On a genetic level, distinct stressors generally result in different selection pressures on distinct gene groups (Lotterhos et al., 2018). The relation between adaptive genes and climate drivers gives information on existing climate adaptations, but when the climate changes it is necessary to know the mismatch between the current and required genomic composition to thrive under the novel conditions. Maladaptation to the anticipated future climate (i.e. genetic offset) can be determined as the shift in the adaptive genetic component required to match the expected climatic changes (Capblancq et al., 2020). Populations with high genetic offset thus require a high (genetic) adaptive capacity to deal with climate change, here defined as the climate‐adaptive genetic variation which determines the potential of populations to change the phenotypic expression of functional traits to environmental change. When the genetic offset is integrated with the adaptive capacity it is possible to predict the sensitivity to climate change of specific populations. Knowledge of the adaptive capacity as well as the degree of maladaptation to climate is a major prerequisite for assisted migration to succeed. Maladaptation to environmental stressors other than climate is another major, but frequently overlooked, determinant of the success of assisted migration (Fitzpatrick & Keller, 2015; Rellstab et al., 2021). Even though some studies integrated the adaptive capacity, genetic offset to climate change, and signatures of maladaptation to environmental stressors in tree species (Jia et al., 2020; Martins et al., 2018; Rellstab et al., 2016), we are unaware of studies that evaluated these factors in forest herbs in order to assess the potential and risks of assisted migration and other potential mitigation strategies. This is essential as forest herbs might experience a reduced amount of evolutionary pressures to climatic factors due to the micro‐climatic buffering capacity of forest ecosystems (Zellweger et al., 2020) and an increased amount of other local adaptation drivers (Baeten et al., 2015; De Kort et al., 2020; Van Daele et al., 2021).

Here, we aimed to assess signatures of selection (allele frequencies deviating from neutral expectations as a consequence of selective pressures imposed by the environment) and climate change sensitivity (as determined by the genetic offset and the genetic adaptive capacity) in *Primula elatior*, a self‐incompatible herb species representative for European moist deciduous forests. Due to its limited dispersal capacity, *P. elatior* is unlikely to track the shifting climate (Honnay et al., 2002; Van Daele et al., 2021; Whale, 1983), rendering its persistence under climate change predominantly dependent upon its adaptive capacity (Bussotti et al., 2015). We sampled 29 *Primula elatior* populations along a large latitudinal gradient to generate single‐nucleotide polymorphisms (SNPs) frequency data, using a genome‐skimming approach (Wessinger et al., 2018). We aimed to (i) identify the population structure and genome‐wide signatures of selection; (ii) examine whether climate is the predominant determinant of adaptive signatures using environment‐association and gene ontology (GO) enrichment analysis; and (iii) quantify the sensitivity of populations to predicted climate change based on their adaptive potential and on the genetic offset.

## 2 METHODS

### 2.1 Study species and data collection

*Primula elatior* subsp. *elatior* mainly occurs in oak or oak‐hornbeam forests in sub‐Atlantic and continental Europe and its distribution ranges from southern France to Northern Denmark (Leuschner & Ellenberg, 2017). *P. elatior* is highly dispersal‐limited due to the absence of morphological seed dispersal adaptations and has specific germination requirements (Taylor et al., 2008). Its flowers exhibit heteromorphic reciprocal herkogamy and the species is self‐incompatible (Keller et al., 2016). Pollen flow is highly limited and rarely exceeds 150 m (van Rossum et al., 2011).

**FIGURE 1:**
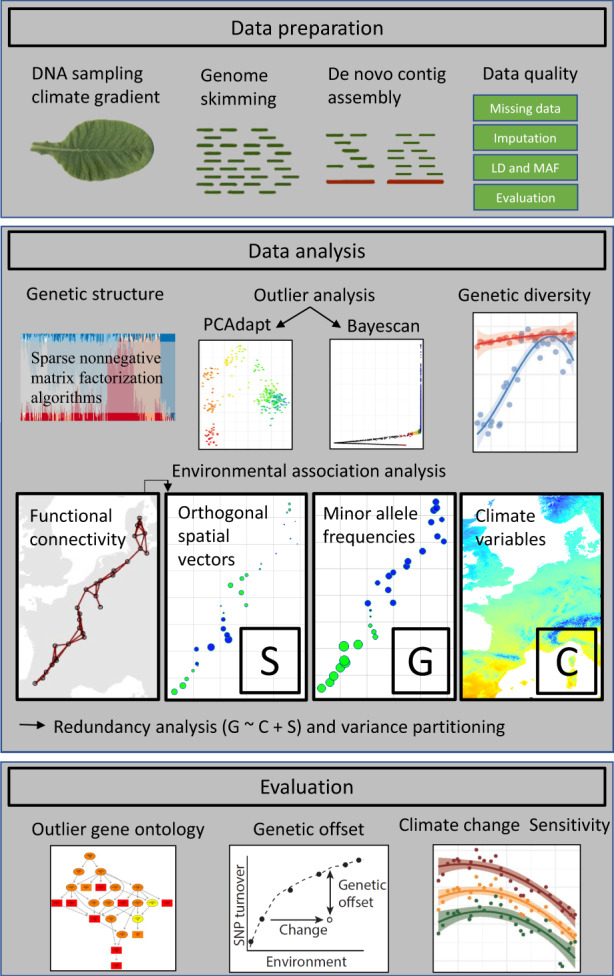
Workflow diagram

**FIGURE 2:**
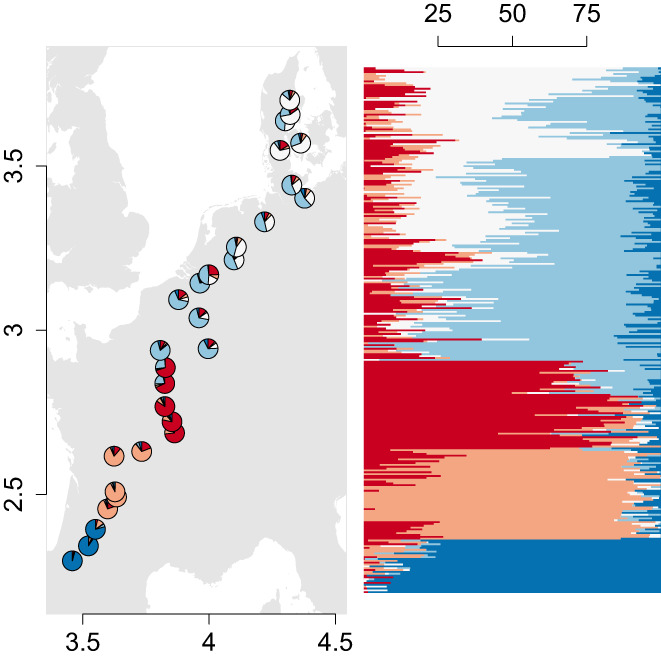
A geographic representation of *Primula elatior* population admixture proportions (left) and individual ancestry coefficients (right) along the range (from southern France up to north Denmark). The longitudinal and latitudinal scale of the distribution range in Europe (left) is indicated in meters × 10^6^ (Coordinate system: LAEA89). The admixture proportions are illustrated as percentages for each individual (*N* = 285).

To capture genome‐wide signatures of selection, we sampled leaf material from 29 large populations (number of flowering individuals ranging from 430 to >1000 individuals) along a latitudinal gradient (c. 1645 km), largely covering the extent of the species' distribution (Figures 1 and 2). Leaf material from nine to 10 individuals at each site, spaced >100 m apart, were sampled and stored in silica gel, mounting to a total of 285 sampled individuals. Total genomic DNA was isolated from dry leaf tissue using a Plant DNA Extraction Kit (Norgen Biotek, 2015).

### 2.2 Genome skimming

#### 2.2.1 Data preprocessing

All libraries for each sequencing lane were demultiplexed using the Illumina ‘bcl2fastq v.1.8.4’ software (Illumina, 2013). One mismatch or missing data point was allowed in the barcode read (5,343,298 ± 1,598,102 average total reads per sample ± SD). Sequencing adapter remnants were clipped from all raw reads and reads with a final length < 20 bases were discarded (2,671,608 ± 799,040 average quality trimmed read pairs per sample ± SD). Adapter‐clipped reads were quality trimmed by removing sequencing errors and trimming was focused on the 3′‐end to get a minimum average Phred quality score of 10 over a window of ten bases (2,512,362 ± 747,790 average adapter clipped read pairs per sample ± SD). Merged quality trimmed reads were error corrected (201,600,000 total read pairs) using ‘Musket v.1.0.6’ with a 21 k‐mer size for correction (Liu et al., 2013). Furthermore, digital normalization of error corrected reads (148,503,886 total reads) were performed with the ‘normalize_by_median.py’ script from ‘khmer v.1.1’ with a k‐mer size of 32 and a coverage cut‐off of 80 (Crusoe et al., 2015).

De novo assembly was performed with the ‘CLC Genomics Workbench v.8.0’ (Qiagen, 2021). Gene discovery was performed with ‘Augustus v.3.1’ (Stanke et al., 2008) on the postprocessed scaffolds. Complementary draft functional annotation of predicted peptides was performed with ‘InterProScan v.5.4‐47.0’ (Jones et al., 2014). InterPro lookups for pathway and GO annotation were performed based on the predicted peptides (Camon et al., 2005). Quality trimmed reads were aligned against *Arabidopsis* as reference genome using ‘BWA‐MEM v.0.7.12’ (Burrows‐Wheeler Aligner; Li, 2013; Li & Durbin, 2009). Variant discovery and genotyping of samples was executed with ‘Freebayes v.1.0.2‐16’ with a diploid setting and reads with more than two mismatches were excluded from the dataset (Garrison & Marth, 2012). Annotations of variant effects on annotated genes and transcripts were performed using ‘SnpEff v.4.31’ (Cingolani et al., 2012). The predicted genes and transcripts from the genome annotation were used to predict downstream functional effects of the variants.

#### 2.2.2 Data filtering, imputation and uncertainty

For the filtering of bi‐allelic SNPs, a minimum read depth of 5 per SNP and a minimum allele frequency across all samples of 5% (Min. MAF = 0.05) were used as threshold. Furthermore, genotypes that were not observed in at least 10% of samples (i.e. at least 29 samples) were removed from the dataset. We selected SNPs with less than 50% missing data within each of at least 11 populations. We made five subsets to maximize the number of SNPs, depending on the amount of populations in which a SNP with less than 50% missing data occurred: all 29 populations (3773 SNPs with 3.9% missing data), 25–28 populations (2638 SNPs with on average 21.7% missing data), 20–24 populations (2760 SNPs with 30.5% missing data), 16–19 populations (1005 SNPs with 34.7% missing data) and 11–15 populations (973 SNPs with 42.0% missing data). This selection procedure resulted in a total of 11,149 SNPs with 20.8% missing data for downstream analysis.

To quantify potential library effects, which may result from sequencing errors in techniques with limited sequencing depth (Mastretta‐Yanes et al., 2015; O'Leary et al., 2018), we sequenced three duplicate samples in order to determine the SNP sequencing error rate for each SNP (73.1% of SNPs were sequenced at least twice). To determine library effects on downstream analyses, three increasingly restrictive SNP matrices, based on the SNP sequencing errors, were constructed (Appendix S1.1, Table S1). First, SNPs that were erroneously sequenced in two out of the three duplicated samples were excluded from the primary dataset (805 SNPs excluded, mean ± SE of the false discovery rate (FDR) = 21.8 ± 0.4). For a second dataset we used the SNPs with calls in only two of the duplicated samples and excluded SNPs that were erroneous in one of these samples (1676 SNPs excluded, mean ± SE of the FDR = 12.4 ± 0.2%). For a third dataset, only SNPs that had no sequencing errors were retained (3945 SNPs excluded, FDR of 0%). Comparative analysis of each SNP matrix against this putatively error‐free dataset allowed assessing the impact of library effects on the results. We found that the genetic diversity and signatures of selection were very similar between datasets (Appendix S1.1, Figure S1) and therefore the SNP dataset with a FDR of 21.8 ± 0.4 was used for all downstream analyses.

The datasets were imputed for analyses that cannot handle missing data (Bayescan, RDA and sNMF and gradient forest). Specifically, the VCF data format was transformed to the genlight data format (containing alternative alleles 0;1;2) and the imputation was based on the rounded population mean. Some SNPs still contained missing data because they had a high missing data percentage on a population level but a low missing data percentage over the whole range (*N* = 279). The specific SNPs in these populations were then imputed based on a mean of three distinct regions (based on the sNMF analysis described in Section 2.3). The mean imputation uncertainty of the final dataset was 3.7% (Appendix S1.2, Figure S2, Tables S2 and S3). Finally, we determined the FDR on a SNP level for each subsequent analysis, by repeating each methodology with distinct parameters (see Sections 2.4 and 2.5 for details), to estimate the cumulative uncertainty of climate‐adapted SNPs.

### 2.3 Population genomics

We combined two methods to identify loci that deviate significantly from background genetic structure, and are therefore assumed to reflect a locus‐specific imprint of diversifying selection. First, using ‘PCAdapt v.4.3.3’ in R (Luu et al., 2017), the K number of principal components were selected based on scree plots and the amount of clustering when PC axes were compared. PCAdapt can avoid problems related to linkage disequilibrium (LD) with a clumping strategy, and less important SNPs were removed based on a window radius of 200 (distance between two SNPs on the same gene) and a squared correlation threshold of 0.1. The mean length of all predicted genes (245,806) was 309.8 ± 254.2 and 330.8 ± 335.6 in the final dataset. A radius window of 200 removed most detectable LD and results were highly similar compared to larger window sizes (Appendix S1.3, Figures S3 and S4). When all five SNP subsets were evaluated with PCAdapt, a total of 3143 SNPs (28.2% of the filtered dataset) were discarded due to LD. Second, this dataset without LD was used in ‘BayeScan v.2.1’ (Beaumont & Balding, 2004), which relies on a Bayesian approach that estimates the posterior probability that a given locus is under selection, thus reducing false positives under a variety of demographic scenarios (Foll & Gaggiotti, 2008). Because we utilized multiple smaller subsets, we designated prior odds of 10 for the neutral model, which correspond to the outlier ratio of PCAdapt in subset 1 (11.5%). A total of 100 pilot runs with a length of 5000 were executed, followed by 100,000 iterations with a burn‐in length of 50,000 (Lotterhos & Whitlock, 2014). The FDRs were determined based on q values and ranged from 0.1% to 50%, with a precision of 0.1%. Deviation from neutrality can be determined based on the locus‐specific effect (alpha parameter in Bayescan), where a positive alpha value indicates diversifying selection and a negative value indicates balancing or purifying selection (Foll & Gaggiotti, 2008). Considering our focus on climate change sensitivity, only outliers under diversifying selection, as determined by the locus‐specific effect in Bayescan (alpha), were evaluated further.

Individual ancestry coefficients were estimated based on sparse non‐negative matrix factorization (sNMF) algorithms (Frichot et al., 2014), using the R package ‘LEA v.3.4.0’ (Frichot & Franc, 2015). SNPs under LD were removed based on the retained SNPs after the PCAdapt analysis. The number of genetic clusters (*K*) tested ranged from 1 to 25 for the first subset and 1 to 15 for subset 2–4. The optimal *K* was selected based on the minimum cross‐entropy of each run. Each run was executed with 10 replicates per *K*, 200,000 iterations and a regularization parameter of 10. Statistical estimates of ancestry proportions were determined based on the sNMF models and depicted graphically.

### 2.4 Environmental association analysis

To identify genetic variation that is linked to climatic variation while accounting for neutral genetic structure shaped by the spatial organization of our populations, we performed a series of redundancy analyses (RDAs) using the R package ‘Vegan v.2.5‐7’ (Orsini et al., 2013). Specifically, a matrix of Hellinger‐transformed minor allele frequencies for each population was analysed against climatic and geographic variables to identify potential drivers of adaptation. Bioclimatic variables (19), with a resolution of 30 arcseconds (~1 km^2^), were used to determine outliers related to climate (Fick & Hijmans, 2017). To distinguish climate effects from spatial effects, we calculated Moran's eigenvector maps (MEMs) based on a spatial weighted matrix of the sampling sites using the R package ‘Adespatial v.0.3‐14’ (Dray et al., 2021). We used a minimum spanning tree (gb‐MEM) to select relevant Euclidian distance connections between populations and converted these to a neighbourhood matrix (Appendix S2, Figure S5). Incorporating the functional connectivity of dispersal modes in MEM, increases the biological realism of spatial vectors (Bauman, Drouet, Fortin, & Dray, 2018; Ver Hoef et al., 2018). Therefore, the cumulative landscape resistances between plots (Van Daele et al., 2021), based on the resistance for dispersal of land‐use, distance to rivers and elevation (*R* ^2^ marginal = 0.76, R^2^ conditional = 0.92), were taken into account using a binary coding scheme following the recommendations of Bauman, Drouet, Dray, and Vleminckx (2018), Bauman, Drouet, Fortin, and Dray (2018). The resulting spatial weights matrix was used to calculate the orthogonal spatial vectors with a positive autocorrelation selection rule (broad spatial clustering; Dray, 2011). The eigenvector computation of the double centred spatial weighting matrix, based on isolation by resistance (IBR), resulted in 14 MEMs with distinct spatial scales. The corresponding eigenvalues are linearly related to Moran's index of spatial autocorrelation. To select relevant spatial scales we performed a priori forward selection of the spatial eigenvectors (MEM) with the first principal component (PCA) of the Hellinger‐transformed minor allele frequencies as response (Bauman, Drouet, Dray, and Vleminckx (2018)), using the mem.select function in ‘Adespatial v.0.3‐14’ with the adjusted *R* ^2^ as discriminator for each subset (Dray et al., 2021). The optimized selection of MEMs relative to the SNP frequencies (based on the adjusted *R* ^2^) resulted in seven significant MEMs for the SNPs in subset 1 (global *R* ^2^ adj. = 22%, *p* = <0.001). The third (*R* ^2^ adj. = 9%, *p* = 0.001) and second (*R* ^2^ adj. = 1.7%, *p* = 0.001) MEM were highly correlated to temperature (*r* = −0.73) and precipitation (*r* = 0.68, Appendix S2, Table S4) relatively, and were therefore excluded for further analysis. The three most significant MEM (#4, #1 and #5 based on eigenvalue ranking) were included as spatial IBR variables in the RDA analyses (Appendix S2, Figure S6).

To select relevant climate variables we used forward and backward selection of partial RDA (pRDA) models (Borcard et al., 1992) with the MEM as conditional variables, calculated with the ordistep function of the ‘Vegan v.2.5‐7’ package in R (Oksanen et al., 2013). Potential explanatory candidate variables were the annual mean temperature (bio 1), max. Temperature of warmest month (bio 5), mean temperature of warmest quarter (bio 10) and precipitation seasonality (bio 15). The temperature variables (bio 1, 5 and 10) were correlated (*r* > 0.6) and were therefore evaluated separately with precipitation seasonality as second climate variable. Resulting models were highly similar due to the high correlation of temperature variables and only the model with the highest explanatory power (*R* ^2^ adj.), which contained max temperature of warmest month and precipitation seasonality as fixed variables (Appendix S2, Figure S7), were retained for further analysis (*R* ^2^ adj. Was 8.9 ± 2.7 for bio 5 compared to 8.5 ± 2.8 for bio 1 and 8.8 ± 2.6 for bio 10). Potential confounding effects between climate and geography were further evaluated with variation partitioning. The FDRs were determined based on q values and ranged from 0.1% to 50%, with a precision of 0.1%. Only outliers under diversifying selection, as detected by Bayescan, were evaluated further.

To obtain insights in the function of outlier loci, we conducted a test for enrichment of GO terms related to outliers (Bayescan, PCAdapt and pRDA) under diversifying selection with <5% FDR. We considered significant GO terms of biological processes with a *p* value lower than 0.05, as detected by the Kolmogorov–Smirnov statistic with classic and elim algorithms (Alexa et al., 2006), and the Fisher statistic with classic and parent–child algorithms (Grossmann et al., 2007). A total of 264 genes in our gene universe had GO functions, and detection analyses of significant GO terms were executed with the R package ‘topGO v.2.44.0’ (Alexa & Rahnenfuhrer, 2021).

### 2.5 Predicting the sensitivity to climate change based on the genetic offset and the adaptive capacity

To assess the adaptive capacity of *P. elatior* across the latitudinal sampling gradient, we modelled the expected heterozygosity (*H*_e_) against latitude (LAEA 89 as coordinate system), a quadratic latitude term to model nonlinear effects, outlier as a binary factor (neutral vs. adaptive), and interactions between latitude and the outlier term (*H*_e_ ~ Latitude × outlier + Latitude^2^ × outlier), using a general linear model. Residuals were normally distributed and no visual heteroscedasticity was detected. Least‐square‐means and pairwise comparisons of outlier genetic diversity with neutral genetic diversity were evaluated with the ‘emmeans v.1.6.3’ package in R (Lenth, 2021). Effect sizes were calculated with the ‘rsq v.2.2’ package (Zhang, 2021).

To calculate the genetic vulnerability to future climatic conditions we calculated the genetic offset with ‘gradientforest v.0.1‐32’ in R, which has shown experimental support for genomic predictions (Fitzpatrick et al., 2021). The gradient forest algorithm calculates the genetic turnover, which reflects the magnitude of genetic distance of many putatively adaptive candidate loci along multiple environmental and geographical gradients (Capblancq et al., 2020; Fitzpatrick & Keller, 2015). The genetic offset reflects the overall genetic distance from the future genetic composition that is required to maintain the current gene–environment relationships (Vanhove et al., 2021). The inherent complexities involved in the calculation of the genetic offset require careful consideration of the potential caveats associated with gradient forest models (Láruson et al., 2022). When genetic drift and migration are not in equilibrium, changes in allele frequencies could reflect genetic drift rather than adaptive signals. Furthermore, smaller populations could exhibit greater signatures of genetic drift compared to larger populations. This could result in population structure gradients that align with environmental gradients. To limit the confounding effects of neutral demography, we selected only large populations (430 to >1000 individuals; see Section 2.1) and evaluated neutral and adaptive outliers separately. Furthermore, a migration‐drift equilibrium could here be assumed due to an observed linear decrease in the genetic similarity at the higher end of the log_10_‐transformed effective resistance, as determined by the isolation‐by‐resistance relationship (Van Daele et al., 2021; Van Strien et al., 2015). Another caveat is that multiple nonlinear environmental gradients could confound the relationship between fitness offset and genetic offset (Láruson et al., 2022). However, our choice to sample along a latitudinal gradient with a relatively linear temperature cline (Figure S7) should have minimized impacts of nonlinear environmental gradients on our findings. Finally, in gradient forest algorithms the current allele frequencies are assumed to reflect the species adaptive optimum. This is caused by the inherent positive genetic offset, regardless of the direction of change (increase or decrease) in allele frequencies across a gradient. The selected large populations, likely migration‐drift equilibrium, and relatively linear temperature clines, are here assumed to have resulted in a stable genomic architecture maintaining associations between fitness and the adaptive gene–environment. This should have produced reliable genetic offset metrics and the corresponding estimation of maladaptation to climate change.

The models were constructed with the Hellinger‐transformed minor allele frequencies of partial RDA outliers (climate as fixed and geographic MEM as conditional) and neutral SNPs as response and the partial RDA selected climate and geographic MEMs as explanatory variables. Bioclimatic projections according to three greenhouse gas scenarios, namely RCP 2.6, RCP 4.5 and RCP 8.5 in 2050 and 2070, were used to predict future predicted turnover. The RCP variables were based on averages from 11 general circulation models (Van Daele et al., 2021). The genetic offset was then calculated as the overall Euclidian distance between the current and future predicted genetic turnover of the climate variables (max. Temperature of warmest month and precipitation seasonality). The relation of genetic offset to latitude was modelled with a linear model and a quadratic term (genetic offset ~ latitude + latitude^2^ × scenario + year). Residuals were normally distributed and no visual heteroscedacity was detected.

To evaluate the sensitivity to climate change of *P. elatior* it is necessary to take both the adaptive capacity and the genetic vulnerability into account (Vranken et al., 2021). To this end, a climate sensitivity metric was constructed for each scenario that corrected the genetic offset for the expected heterozygosity of partial RDA outliers in each population:

$$\begin{array}{c} \text{Climate Sensitivity}\\ \;=\text{sqrt}\left(\text{Genetic Offset}\times \left[1–\text{Expected Heterozygosity of climate outliers}\right]\right). \end{array}$$

This formula gives a similar weight to both the expected heterozygosity and genetic offset as the range of both metrics are highly similar (0–0.3). The relation of climate sensitivity to latitude was modelled with a linear model and a quadratic term (genetic offset ~ latitude + latitude^2^ × scenario + year). Residuals were normally distributed and no visual heteroscedacticity was detected.

## 3 RESULTS

### 3.1 Outlier analysis and population structure

**FIGURE 3:**
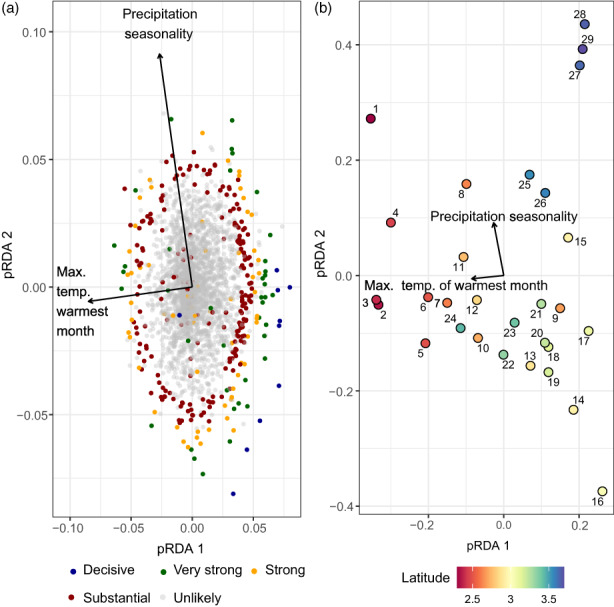
The partial redundancy analysis (pRDA) biplot in panel a illustrates the pRDA scores of the first two axes with *Primula elatior* single nucleotide polymorphisms (SNPs) as response variables, climate as fixed explanatory variables and isolation by resistance (IBR) moran eigenvector maps as conditional explanatory variables. Decisive outliers had <1% false discovery rate (FDR), very strong outliers <5% FDR, strong outliers <10% FDR and substantial outliers <25% FDR. Only decisive and very strong outliers were used for further analysis. The pRDA biplot in panel b displays the pRDA scores of the sampled population sites along the distribution range in Europe. Numbers indicate the plot ID from south (low) to north (high). The latitude colour scheme indicates the latitude in metres × 10^6^ (Coordinate system LAEA89). The statistical evaluation of the pRDA model (Climate | IBR) can be found in Table 1.

With a FDR threshold of 5%, PCAdapt detected 266 outliers (3.4%) under diversifying selection, while 163 outliers (2.1%) were detected with a FDR below 1%, and 107 outliers (1.4%) with a FDR below 0.1%. A large amount of outliers were thus featured by a decisive signature of diversifying selection (Figure 3). Along the first PC axis three clusters could be identified with a clear genetic differentiation between southern, central and north European populations (Appendix S3, Figure S8). The second PC axis mostly differentiated the southern populations. With a FDR below 5%, Bayescan detected 448 (5.8%) outliers under diversifying selection (1.7% under balancing selection) while 305 (3.9%) outliers were detected with a FDR below 1%, and 227 outliers (2.9%) with a FDR below 0.1%. Similar to PCAdapt, most outliers were thus decisive (Figure 3). Together, a total of 172 (2.2%), 102 (1.3%) and 77 (0.99%) diversifying outliers were detected by both methods using a FDR threshold of 5%, 1% and 0.1% respectively.

Inference of the population structure through sNMF analysis resulted in five ancestral clusters when all populations were considered (subset 1). SNPs that did not occur in all populations (subset 2–4) were aggregated in 14 distinct clusters but did not yield additional insights. Southern populations had a low amount of mixture but with three abrupt cluster transitions between populations (Figure 2). Northern populations, on the other hand, had a large amount of mixture and smooth transitions between clusters (Figure 2, Appendix S3, Figure S8).

### 3.2 Unravelling environmental drivers of local adaptation

The included isolation‐by‐resistance MEM with the strongest contribution (MEM 4, *R* ^2^ adj. = 2.8%, *λ* = 3.46, *p* = 0.001) had strong regional eigenvector value turnover in south Europe and central Europe (similar to the dark blue and red genetic turnover in Figure 2; Appendix S2, Figure S6). The second most contributing eigenvector (MEM 1, *R* ^2^ adj. = 2.8%, λ = 3.9, *p* = 0.001) was mostly related to regional eigenvector value turnover in north Germany, and the final included eigenvector (MEM 5, *R* ^2^ adj. = 2%, *λ* = 3, *p* = 0.001) was related to regional eigenvector value turnover in south‐central Europe (similar to the edges of the red cluster in Figure 2).

Climate had more explanatory power on SNP frequency variance than eigenvectors based on IBR in both the RDA and partial RDA models, and the overlap in explanatory power was minimal (Table 1). Most of the variation in the partial climate model, which excluded IBR eigenvector effects (Climate|IBR), was related to the first RDA axis (*σ* ^2^ = 0.011, *F* = 4.60, *p* = 0.001), which mainly represents the maximum temperature of the warmest month (*σ* ^2^ = 0.011, *F* = 4.49, *p* = 0.001). The second RDA axis (*σ* ^2^ = 0.003, *F* = 1.30, *p* = 0.009) was mostly related to precipitation seasonality (*σ* ^2^ = 0.004, *F* = 1.41, *p* = 0.064, residual *σ* ^2^ = 0.057) with the most northern populations and the most southern populations featured by strong precipitation seasonality, and some central European populations by weak precipitation seasonality (Figure 3).

**TABLE 1:** Results of the (partial) redundancy analyses (RDAs) to partition among‐population genetic variation of *Primula elatior* into climate, isolation by resistance (IBR), and their combined effects.

Model	df	Residual df	Variance	Residual variance	*F*	*p*	*R* ^2^ adj. (%)
Climate	2	26	0.02	0.07	3.27	0.001	14
IBR	3	25	0.01	0.07	1.61	0.002	6
Climate + IBR	5	23	0.03	0.06	2.30	0.001	19
**Climate | IBR**	**2**	**23**	**0.01**	**0.06**	**2.95**	**0.001**	**13**
IBR | Climate	3	23	0.01	0.06	1.52	0.001	5
Climate ∩ IBR							1
Total explained							19
Total unexplained							81

*Note*: This is the result table of the first subset as only six out of the 63 outliers were detected in subset 2–5. The partial climate effect (excluding IBR) is depicted in bold.

**FIGURE 4:**
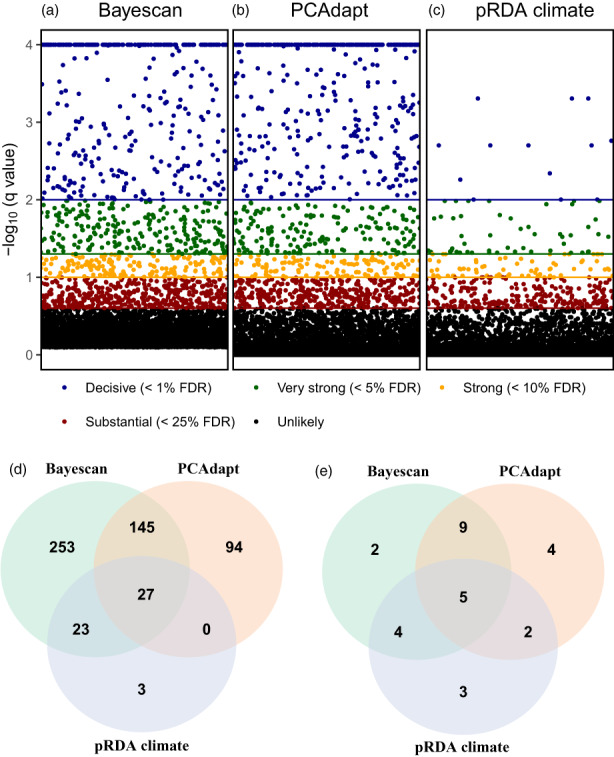
Manhattan plot of Bayescan (a), PCAdapt (b) and partial RDA (c) outlier locus detection analyses of *Primula elatior* across subsets. The legend displays the false discovery rates (FDR) of individual single nucleotide polymorphisms and the horizontal lines with matching colours display the FDR likelihood thresholds. Panel d displays the Venn diagram of detected SNP outliers under diversifying selection as detected by the distinct analyses. Panel e displays the Venn diagram of gene ontology outliers under diversifying selection that were related to biological processes.

The partial RDA detected 63 outliers (0.8%) with a FDR below 5%, which were thus related to climatic clines, and 53 outliers (0.7%; Appendix S4, Table S5) of these were under diversifying selection (as determined by Bayescan). A total of 12 outliers under diversifying selection had a FDR below 1% and three outliers had a FDR below 0.1%. Of the outliers with a FDR below 5%, 50 were also detected by Bayescan and 27 by PCAdapt (of which 21 under diversifying selection). The three methods combined detected 21 outliers under diversifying selection, of which seven were decisive (Figure 4). A total of 448 SNPs (5.6%) had significant signatures of diversifying selection according to Bayescan (FDR of 5%) and 398 SNPs (5%) were not associated with climate. For PCAdapt, a total of 266 (3.3%) outliers under diversifying selection were detected and 239 (3%) of those were not related to climate. A total of 127 SNPs (1.6%) were identified by both methods as diversifying outliers for nonclimatic selection pressures and 111 (1.4%) of these could not be related to IBR patterns (pRDA IBR|Climate) as well.

A total of 22 genes contained signatures of climate adaptation under diversifying selection pressures (Appendix S4, Table S6), six of these genes were linked to a total of 14 GO terms that were significantly enriched for biological functions, and five GO terms were detected by all three outlier detection methods. Most of these significant biological functions were related to metabolic processes and no significant relation to response to stress could be detected (annotated = 5, significant = 0, expected = 0.12, p value KS classic = 0.9). Interestingly, photosynthesis was detected as a significant biological function by Bayescan (annotated = 5, significant = 5, expected = 1.2, *p* value KS classic = <0.001) and PCAdapt (annotated = 5, significant = 4, expected = 0.7, *p* value KS classic = 0.001) but not by the partial RDA (annotated = 5, significant = 0, expected = 0.12, *p* value KS classic = 0.128). This could suggest a role for environmental variables other than climate (e.g. photoperiod) as an important driver of selection in *P. elatior*. The relation between significant photosynthetic outlier SNPs and spring trimester direct normal irradiation (Copernicus Atmosphere Monitoring Service, 2020) as a photoperiodicity indicator was evaluated with spearman correlation tests. Frequencies of two SNPs related to photosynthesis had moderate spearman correlation to spring trimester direct normal irradiation (contig_101_20702 = 0.57 and contig_110_5079 = −0.47; Appendix S4, Figure S10).

### 3.3 Sensitivity to climate change across a latitudinal gradient

**FIGURE 5:**
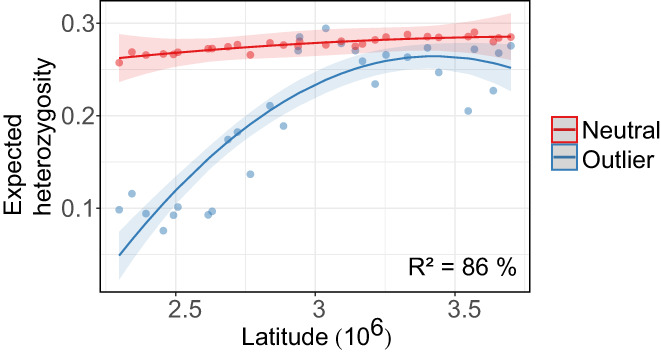
Expected heterozygosity (*H*_e_) of *Primula elatior* across the latitudinal distribution range. *H*_e_ is illustrated for climate outliers (blue), identified using RDA while partialling out the effect of isolation by resistance moran eigenvector maps, and neutral SNPs (red). The distinction in the model fit between outlier *H*_e_ and neutral *H*_e_ was addressed by an interaction effect of a binary outlier term with latitude and quadratic latitude respectively: *H*_e_ ~ Latitude × outlier + Latitude^2^ × outlier.

The genetic diversity (*H*_e_) of SNP frequencies under diversifying selection driven by climatic clines as predicted by our general linear model (estimated marginal means ± SE = 0.23 ± 0.007) was significantly lower (*Z* ratio = 4.6, *p* < 0.001) than the neutral genetic diversity (estimated marginal means ± SE = 0.28 ± 0.007), particularly towards the south of the distribution (outlier:latitude^2^; *F* = 14.6, *p* < 0.001; Figure 5). Predicted genetic diversity was also lower for Bayescan outliers (*Z* ratio = 19.57, *p* < 0.001) and PCAdapt outliers (*Z* ratio = 5.16, *p* < 0.001).

**FIGURE 6:**
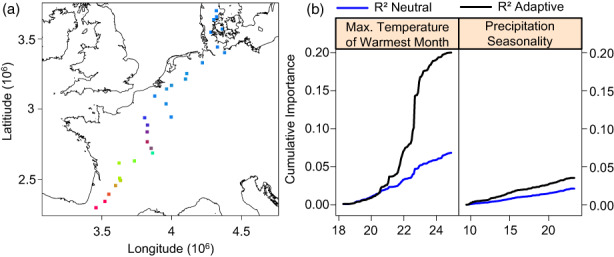
SNP turnover in allele frequencies (panel a) of *Primula elatior* as predicted by the gradient forest model with the climatic partial RDA outliers as response. Principal component analysis (PCA) was used to reduce the genetic turnover of each predicted variable into three principal components that were each assigned a RGB colour. The colour code does not have a scale but similarity between colours indicates the relation of predicted adaptive genetic frequencies between populations. The relation of the cumulative importance (*R* ^2^) of neutral and adaptive SNPs to explanatory climate variables can be found in panel b.

**FIGURE 7:**
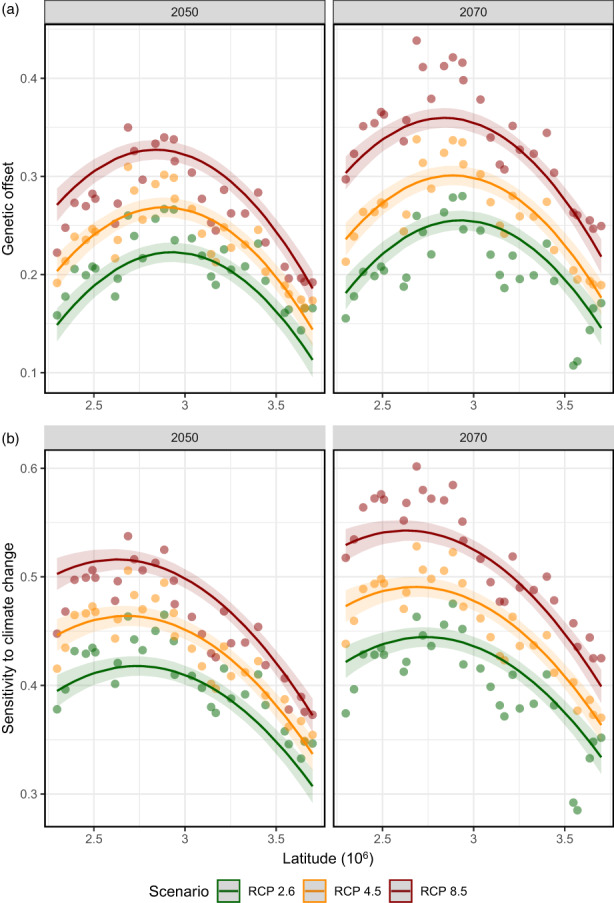
The genetic offset and climate sensitivity of *Primula elatior* climate outlier SNPs (Climate | IBR; Table 1; Figures 3 and 4) as predicted by the gradient forest algorithm. The genetic offset reflects the overall Euclidian genetic distance between the current adaptive genetic turnover (Figure 6) and the predicted adaptive genetic turnover in 2050 (left) and 2070 (right) when gene–environment relationships would be maintained. Higher genetic offset values indicate an increased maladaptation to climate change. The selected bioclimatic variables that determined the genetic offset are the max. Temperature of the warmest month and precipitation seasonality. Climate projections were based on three greenhouse gas scenarios, namely RCP 2.6, RCP 4.5 and RCP 8.5 in 2050 and 2070 (average from 11 general circulation models). The climate sensitivity was calculated as the genetic offset weighted by the adaptive capacity (*H*_e_ of climate outliers): sqrt(Genetic Offset × [1–*H*_e_]). Both panels were based on the following linear models: response ~ latitude + latitude^2^ × scenario + year).

Our gradient forest algorithm predicted strong genetic turnover of climate related SNP outliers around the 22°C maximum temperature of the warmest month threshold (light blue to dark blue crossover in Figure 6), with three abrupt transitions in southern Europe (Figure 6, Appendix S3, Figure S8). The genetic offset was highest in central France around the 22°C threshold and then gradually decreased towards the north (Figure 7; *R* ^2^ = 80.5%), and was predicted to be significantly higher in 2050 under RCP 8.5 (estimated marginal mean = 0.32, LCL = 0.31, UCL = 0.33) compared to RCP 4.5 (EMM = 0.27, LCL = 0.26, UCL = 0.28, *Z* ratio = −11.1, *p* = <0.001) and RCP 2.6 (EMM = 0.22, LCL = 0.21, UCL = 0.23, *Z* ratio = −18.8, *p* = <0.001). Furthermore, the genetic offset was predicted to significantly increase in 2070 compared to 2050 (*Z* = 7.4, *p* = <0.001).

The climate outliers of the eight most southern populations had a relatively low genetic offset (Figure 7, red to green in Figure 6), but also had significantly reduced genetic diversity for climate outliers (Figure 5). Consequently, the overall sensitivity to climate change (as determined by genetic diversity and genetic offset) was high in the south and only starts to decrease in northern France (*R* ^2^ = 83.3%). The sensitivity to climate change was predicted to be significantly higher in 2050 under RCP 8.5 (EMM = 0.5, LCL = 0.49, UCL = 0.51) compared to RCP 4.5 (EMM = 0.45, LCL = 0.44, UCL = 0.46, *Z* ratio = −10.03, *p* = <0.001) and RCP 2.6 (EMM = 0.41, LCL = 0.40, UCL = 0.42, *Z* ratio = −18.94, *p* = <0.001). Furthermore, the sensitivity to climate change was predicted to significantly increase in 2070 compared to 2050 (*Z* = 6.95, *p* = <0.001).

## 4 DISCUSSION AND CONCLUSION

Using population genomic analyses, we detected ample genetic outliers related to local adaptations along a latitudinal gradient of *Primula elatior* populations. Furthermore, strong genetic differentiation in the south of the distribution range was detected. Gene–environment association analyses uncovered local adaptations to clines of maximum temperatures and to a lesser extent the precipitation seasonality. Most of the outliers detected by population genomic analyses were, however, not related to climate. Biological functions related to climate outliers were generic metabolism functions and no adaptations to drought were detected. Based on current gene–environment associations and predictive machine‐learning‐based modelling, we identified a high sensitivity of *P. elatior* to climate change in the south and centre of the distribution range, relative to more northern populations. However, because detected preadapted alleles to climate change were limited and because target populations in the north of the range were characterized by relatively high adaptive potential, our findings suggest that *P. elatior* would not benefit from south‐to‐north translocation. Moreover, the important contribution of nonclimatic signatures of local adaptation (e.g. photoperiod), indicates a high risk of maladaptation when southern and central populations would be translocated to northern locations. Our integrated analyses provide a first insight on the potential evolutionary constraints that could hamper adaptation to climate change in forest herbs and could limit the potential of assisted migration as a mitigation strategy.

### 4.1 Population structure and signatures of selection

The population genomic scans of *Primula elatior* uncovered considerable diversifying selection (3.4–5.8%) over the entire latitudinal range. The population structure analysis (sNMF) indicated major clustering with sharp genetic turnover between southern regions. On the other hand, central and north European populations had a large degree of mixture, which is consistent with the idea of postglacial colonization of northern Europe from multiple southern refugia (Figure 2; Appendix S3, Figures S8 and S9; Hewitt, 1999; Sommer & Zachos, 2009). The IBR spatial scale (MEM) that contributed most to the genetic composition closely matched the genetic turnover patterns of the population structure (Figure 2; Appendix S2). This indicates that the integrated IBR patterns were good indicators for the demography and gene flow (Hoban et al., 2016), which explained 5% of allele frequencies (Table 1).

The strong genetic differentiation along the range (Figure 2) favours the use of local genetic material for restoration purposes (Breed et al., 2018; Broadhurst et al., 2008). However, rapid climate change requires considerable adaptive shifts that may not be achieved without assisted gene flow. Here, we found considerable signatures of diversifying selection across the latitudinal gradient, pointing to the occurrence of alleles preadapted to a warming climate somewhere along the gradient. Climatic clines explained a considerable amount of genetic variation between populations (13%), but only a small amount of SNPs were significant outliers under diversifying selection that were likely to be driven by climatic selection pressures (0.7%). Furthermore, most of the outliers were biological functions related to metabolic processes and no adaptations to drought or heat stress were detected. However, it has to be noted that the stringent selection procedure, which was used to avoid uncertainties in the data analyses, could have eliminated potentially informative alleles on drought and heat resistance. Complementary sequencing techniques with higher read depth, preferably combined with common garden experiments, could potentially uncover overlooked drought resistance signatures, which are often characterized by a complex polygenic architecture. Nevertheless, growth regulation in response to seasonal factors is known to be important in perennials (Wingler, 2015) and perennial herbs generally have a greater capacity for temperature acclimation than annuals (Yamori et al., 2014). Temperature regulation results in altered carbon dynamics and generates sugar signals that further modulate metabolic pathways involved in biosynthetic and catabolic processes. These metabolic processes can in turn result in local adaptations of relative growth, caused by resource allocation trade‐offs in acclimation regulation processes (Gray & Brady, 2016; Wingler, 2015). Furthermore, a longer growing season length and thus a higher resource availability in southern populations may strengthen local adaptation towards greater allocation to growth and reproductive traits within a species (López‐Goldar & Agrawal, 2021). The lack of climate SNPs associated with drought stress could be related to the microclimatic buffering capacity of forest canopies and riparian zones (De Frenne et al., 2019; Moore et al., 2005).

Although we detected quite some climate SNPs (29%), most of the adaptive outliers under diversifying selection could not be linked to climate (71%), indicating that environmental factors other than climate cause stronger signatures of adaptive divergence along the latitudinal gradient. Interestingly, we detected multiple significant photosynthetic adaptations (5) unrelated to climate. As we discovered correlations between some outlier SNP frequencies and spring trimester direct normal irradiation, it is likely that photosynthetic adaptations are related to solar irradiation. Photosynthetic acclimation is particularly important in perennial, long‐lived species that experience a high seasonality during their lifespan (Yamori et al., 2014). *P. elatior* is highly dependent on the short light phase before tree canopy leaf development in spring (Baeten et al., 2015; Taylor et al., 2008) and photosynthetic adaptations may have a strong relation to phenological adaptations and growth regulation (Poorter et al., 2009; Rothstein & Zak, 2001). However, further research is needed to disentangle the intricate relationships between plant metabolism, photosynthesis, and local adaptation to climatic factors.

### 4.2 Climate sensitivity

Forest herbs lacking adaptations for long‐distance dispersal are estimated to lose genetic diversity at a faster rate under an increasingly changing climate (Alsos et al., 2012). Without adaptive evolution, *Primula elatior* is expected to lose over half of the total distribution area by 2050 due to climate change and dispersal limitation (Van Daele et al., 2021). Furthermore, southern populations are expected to be most affected and therefore populations will be highly dependent on their adaptive capacity. The potential of *P. elatior* to adapt to the changing climate will depend on the genetic offset of climate outliers and the adaptive genetic diversity, together shaping its climate sensitivity. We found that the cumulative importance of climate outliers sharply increased between a max. Temperature between 22°C and 23°C, which corresponds to a sharp increase of the adaptive genetic turnover in Central France. The sharp increase in adaptive genetic turnover is likely related to a combination of climate selection pressures (Appendix S2, Figure S7) and low gene flow in these regions (Appendix S3, Figure S9). When environmental change increases, gene flow is needed to introduce preadapted alleles and enable local adaptation under the changed conditions (Blanquart et al., 2013). However, the expected climate shift in this region resulted in a high genetic offset in central Europe and the low migration rate due to dispersal limitations are unlikely to alleviate climate change effects (Van Daele et al., 2021). Overall, southern and central European populations are more sensitive to climate change than northern populations. It is however important to take into account that the appropriate application of the gradient forest algorithm is still under active development and that the genetic offset could be confounded by the polygenic nature of drought resistance (Láruson et al., 2022). Furthermore, the potentially incomplete depiction of the genomic architecture and unverified relationship between the adaptive genetic offset and the adaptive capacity could have influenced the climate sensitivity curve. Further research is needed to evaluate the empirical relationship of the calculated climate sensitivity and plant fitness under climate change. Nevertheless, the low adaptive capacity, likely maladaptation of southern genotypes to longer photoperiods and other local conditions, and potential outbreeding effects could jeopardize successful assisted migration from southern to central and northern European regions. Therefore, conservation measures should prioritize preserving or improving (meta‐)population stability trough ecological restoration of the habitat quality and ecological connectivity. Additional measures could include admixture provenancing within population clusters, characterized by low genetic turnover and a wide selection from various environments (Figure 2; Breed et al., 2013), to improve (south/centre) and maintain (north) the adaptive capacity across the range (Vergeer et al., 2004; Whiteley et al., 2015). After careful monitoring of the admixture provenancing, it could then be decided to introduce offspring into more central European populations to alleviate their high genetic offset. Finally, the high adaptive genetic diversity in northern populations and the low climate offset could enable successful range expansions to projected suitable habitat in Scandinavia. As many perennial forest herbs exhibit similar dispersal modes and life history strategies (Verheyen et al., 2003), experience buffered climatic conditions (Zellweger et al., 2020), and are generally sensitive to photoperiodism (Flynn & Wolkovich, 2018), it is likely that evolutionary constraints may impact the efficacy of assisted migration across forest species. Further research can shed light on the general tendency of maladaptation and climate sensitivity in forest species.

## CONFLICT OF INTEREST

The authors declare no conflict of interest.

## Supporting information


Appendix S1–S4

## Data Availability

Genomic sequencing data (VCF) and georeferenced sampling locations are deposited in a Dryad data repository named ‘Genome skimming SNP dataset of *Primula elatior* individuals along a latitudinal cross‐section of the distribution range’ (Van Daele et al., 2022) and can be accessed through the following link: https://doi.org/10.5061/dryad.b5mkkwhg0. BENEFIT‐SHARING: Genetic data were collected in accordance with the Nagoya protocol on access to genetic resources (NOR: TREL1902817S/128). Benefits from this research accrue from the sharing of our data and results on public databases as described above.
