# Supplementary material for: Genomic analyses point to a low evolutionary potential of prospective source populations for assisted migration in a forest herb
Source: Evol Appl. 2022 Oct 2;15(11):1859–74. doi: 10.1111/eva.13485 (PMC9679244; doi:10.1111/eva.13485)
Supplement: Supplementary file 1 — Appendix S1–S4 [file EVA-15-1859-s001.docx]

**Supplemental Information for:**

**Genomic analyses point to a low evolutionary potential
of prospective source populations
for assisted migration in a forest herb**

Frederik Van Daele; Olivier Honnay; Hanne De Kort

**Table of Contents:**

| **Appendix S1** | **Quantifying uncertainty in the detection of local adaptation** | Page 3 |
| --- | --- | --- |
| Appendix S1.1 | Library effects | Page 3 |
| Appendix S1.2 | Imputation accuracy | Page 4 |
| Appendix S1.3 | Linkage disequilibrium | Page 4 |
| **Appendix S2** | **Environmental association drivers** | Page 8 |
| **Appendix S3** | **Indications for post-glacial (re)colonization** | Page 10 |
| **Appendix S4** | **SNP and GO ontology outliers** | Page 12 |
| **References** |  | Page15 |

**Table of Figures:**

| **Figure S1** | **Library effects on Bayescan and PCAdapt  outlier analyses, and expected heterozygosity** | Page 4 |
| --- | --- | --- |
| **Figure S2** | **The relation between missing data and  the imputation accuracy** | Page 6 |
| **Figure S3** | **LD pruning effects** | Page 7 |
| **Figure S4** | **Moran's eigenvector map method diagram** | Page 8 |
| **Figure S5** | **Method diagram for quantifying Moran's  eigenvector maps of the isolation by resistance** | Page 9 |
| **Figure S6** | **Eigenvalues of the selected Moran eigenvector maps and the plot scores in geographical space** | Page 10 |
| **Figure S7** | **Distribution of plots and their  relation to the max. temperature of the  warmest month and precipitation seasonality** | Page 11 |
| **Figure S8** | **Biplot of PCAdapt of the first two principal components** | Page 12 |
| **Figure S9** | **The relation between the average  genetic distance and latitude** | Page 12 |
| **Figure S10** | **Relation between irradiance and  photosynthesis SPN frequencies** | Page 16 |

**Table of Tables:**

| **Table S1** | **The distinct datasets used for quantifying  library effects and the sequencing false  discover rate (FDR)** | Page 3 |
| --- | --- | --- |
| **Table S2** | **SNPs affected by imputation and the amount of missing data that was imputed accordingly** | Page 5 |
| **Table S3** | **Mean imputation accuracy and the standard  deviation of specific missing data thresholds** | Page 5 |
| **Table S4** | **Spearman correlation coefficients of climate  and spatial variables** | Page 8 |
| **Table S5** | **Overview of climate outliers and their FDR** | Page 12 |
| **Table S6** | **Significant biological processes of  gene ontology outliers related to climate as determined by the partial RDA** | Page 14 |

# Appendix S1: Quantifying uncertainty in the detection of local adaptation

To quantify the uncertainty for each potential outlier, we calculated the SNP accuracy for each step in the process. First, we quantified the uncertainty related to library effects by evaluating the SNP calling false discovery rates. Second, we determined the uncertainty related to population level imputation accuracies. Finally, we calculated the false discovery rate for each analyses.

## S1.1. Library effects

To take library effects into account (Mastretta-Yanes et al., 2015; O’Leary, Puritz, Willis, Hollenbeck, & Portnoy, 2018), we sampled three duplicate samples and determined the SNP sequencing error rate for each SNP (73.1% of SNPs were sequenced at least two times). The 3003 SNPs that were not called in the duplicate samples were assigned a mean SNP error rate of 26.9 ± 0.4, which corresponds to the SNP error rate of the unfiltered dataset (SNP dataset 0;Table 1). SNPs that were erroneously sequenced in two out of the three duplicated samples were excluded for the primary dataset used in the analyses (SNP dataset 1; Table 1). The SNPs which were called in two out of the three duplicated samples and erroneous in one of these samples were excluded for the second SNP dataset. For the third SNP dataset, only SNPs that had no sequencing errors were retained. Comparative analysis of each SNP matrix against this putatively error-free dataset allowed assessing the impact of library effects on the results. Subsequent analysis for PCAdapt and Bayescan were executed on all three SNP datasets to determine library effects.

Table S1: The calculated false discovery rate (FDR) of SNPs, based on multiple independent sequences of 3 distinct samples. The “SNP dataset” column depict the distinct datasets which were determined by the filtering procedure of the error rate described in S1.1 and chapter 2.2.2. The “Dataset size” depicts the total amount of SNPs in the described dataset. “Evaluated SNPs” depict the amount of SNPs that were called at least two times. “FDR (%)” depicts the calculated mean false discovery rate of SNPs ± the standard error in the duplicated samples. “SNPs excluded” depicts the SNPs that were excluded based on the false discovery rules that determined the distinct SNP datasets (see S1.1 and chapter 2.2.2.). The “dataset size” is determined by the less stringent dataset size (row above) minus the excluded SNPs. SNP dataset 1 is the dataset used for all analyses in the manuscript.

| SNP dataset | Dataset size | #SNPs evaluated | FDR (%) | SNPs excluded |
| --- | --- | --- | --- | --- |
| 0 | 11954 | 8951 | 26.9 ± 0.4 | 0 |
| **1** | **11149** | **8146** | **21.8 ± 0.4** | **805** |
| 2 | 10278 | 7275 | 12.4 ± 0.2 | 1676 |
| 3 | 8009 | 5006 | 0 ± 0 | 3945 |

Library effects on outlier detection, as determined by bayescan and PCAdapt, was negligible up to an uncertainty threshold of 10%. With a threshold higher than 10%, the outlier percentage of bayescan analyses was higher for the datasets with more restrictive filtering (Fig. S1, panel A). With an uncertainty threshold of 5% in bayescan, only 40 outliers were subject to library effects (Fig. S1, panel B). Furthermore, library effects on the expected heterozygosity were negligible as well (Fig. S1, panel C). Therefore, we deemed the results of library 1 as accurate and reliable.

Figure S1: A) Library effects on Bayescan and PCAdapt outlier analyses. Each datapoint displays a separate outlier analysis. The outlier percentage is relative to the amount of SNPs in the respective SNP dataset. The SNP datasets depict the distinct datasets which were determined by the filtering procedure of the error rate described in S1.1 and chapter 2.2.2. The uncertainty threshold corresponds to the false discovery rate threshold that was used in the corresponding analysis. The false discovery rate is defined as the percentage of false discoveries expected in the list of SNP outliers. B) venn diagram of library effects on bayescan results with a false discovery rate threshold of 5%. This includes all outliers. C) Library effects on the population-level expected heterozygosity.


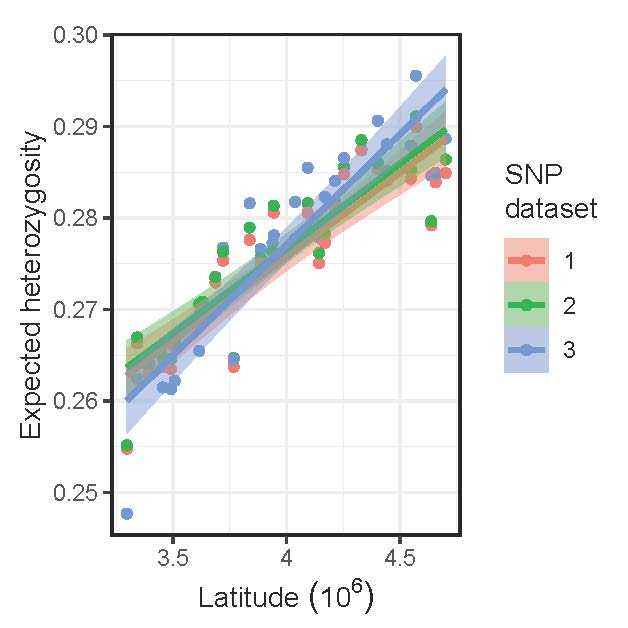

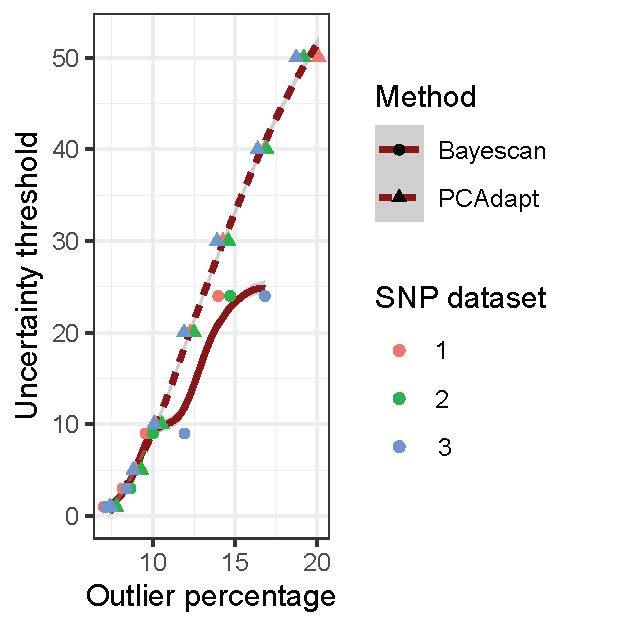

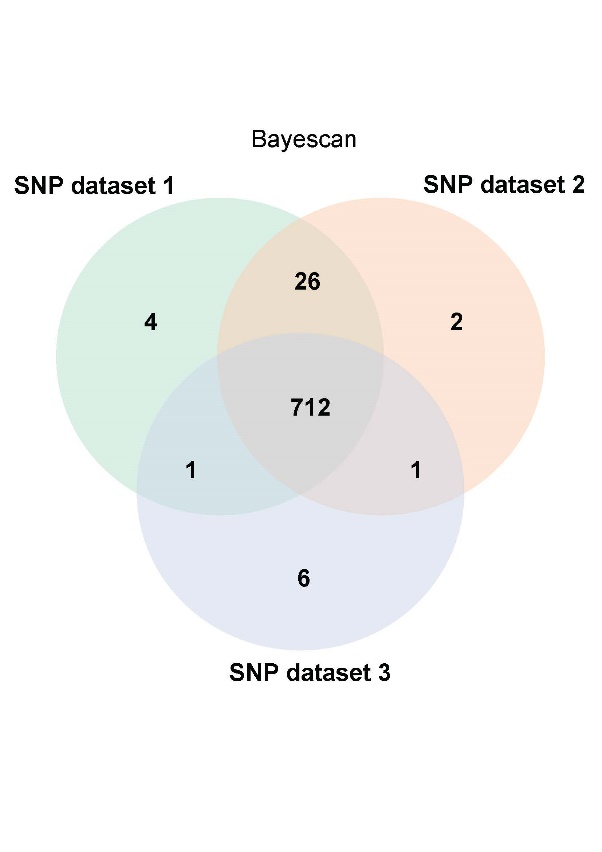


A

B

C

## S1.2 Imputation accuracy

The accuracy for population and region based mean imputation was calculated on a subset without missing data (1906 SNPs) with 100 random imputations for missing data levels ranging from 10 to 100%. After population mean based imputation, some SNPs still contained missing data because they had a high missing data percentage on a population level but a low missing data percentage over the whole range (N=279). Therefore, a region based imputation was executed on the remaining 279 SNPs, which contained missing data ranging from 3.3 to 13.5%. This was based on the individual ancestry coefficients of the sNMF analysis and was calculated as the mean of population 1 to 8 (south), 9 to 13 (central) and 14 to 29 (north). Because the imputation accuracy was highly similar between population imputation and region imputation (<1% difference), we used the population imputation for evaluation. The percentage of missing data within a SNP (Table S1) was then multiplied with the related accuracy (Table S2) to determine the imputation error on a SNP level. The threshold for missing data was determined based on the slope of the imputation accuracy (Fig. S2), which starts to decline around (50%).


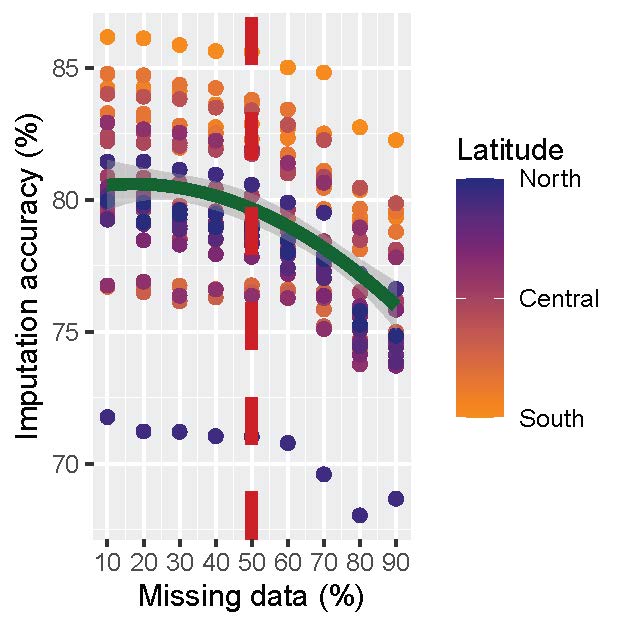


*A)* *Figure S2. The relation between the amount of missing data that was integrated with random permutations (100) in the subset without missing data (1906 SNPs) on a population level. The colour scheme indicates the latitude of specific populations. The green line indicates the quadratic function fit and its confidence interval (grey). The red dotted line indicates the missing data threshold that was permitted in the datasets used in the manuscript. The imputation accuracy is relatively stable when <= 50% (red dotted line) of the data is missing within each population.*

*B) Table S2.* *SNPs affected by imputation and the amount of missing data that was imputed accordingly.*

C) Table S3. Mean imputation accuracy and the standard deviation of specific missing data thresholds (missing).

| Missing data | SNPs imputed |
| --- | --- |
| 0-1% | 1953 |
| 1-10% | 1329 |
| 10-20% | 1701 |
| 20-30% | 2471 |
| 30-40% | 2823 |
| 40-50% | 898 |

| Missing (%) | Accuracy | SD | Imputation error |
| --- | --- | --- | --- |
| 10 | 80.66 | 3.03 | 19.34 |
| 20 | 80.56 | 3.02 | 19.44 |
| 30 | 80.13 | 2.98 | 19.87 |
| 40 | 79.94 | 2.94 | 20.06 |
| 50 | 79.8 | 2.9 | 20.2 |

A

B

C

## S1.3 linkage disequilibrium

PCAdapt can avoid problems related to linkage disequilibrium (LD) with a clumping strategy, removing less important SNPs based on a window radius (distance between two SNPs on the same gene) and a squared correlation threshold (0.1). The mean length of all predicted genes (245,806) was 309.8 ± 254.2 and 330.8 ± 335.6 in the final dataset. To assess the effect of LD pruning on outlier differentiation in PCAdapt we evaluated the difference between no LD pruning, and LD pruning with a window size of 100, 200, 300, 400 and 500 respectively. The majority of SNPs were not affected by LD pruning and were detected irrespective of the LD window size (Fig. S3). To nevertheless avoid LD effects in other analyses as well we applied a radius window of 200, which should remove most LD.


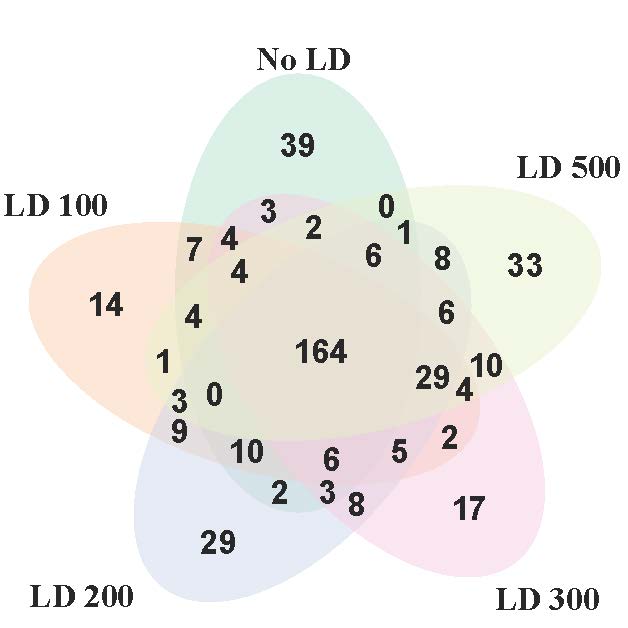


Figure S3: Venn diagram of the linkage disequilibrium pruning effect on SNP outliers, as determined by the LD window size in the PCAdapt procedure.

The LD pruning with a window size of 200 resulted in the visual absence of linkage disequilibrium in index plots of PCAdapt loadings (Fig. S4). When all five SNP subsets were evaluated with PCAdapt, a total of 3402 SNPs (30.5 % of the filtered dataset) were discarded due to LD. Second, this dataset without LD was used in `BayeScan v.2.1´ (Beaumont & Balding, 2004) and redundancy analyses.

Figure S4: LD pruning effects as determined by the PCAdapt analyses. The LD analyses of the distinct subsets display the relation between the index position (contig > FASTA position) versus the PC loadings on the first PC axis. The LD pruning was executed with the pcadapt function in R (Luu et al., 2017) and LD.clumping parameters were set with a LD window of 200 and a correlation threshold of 0.1.


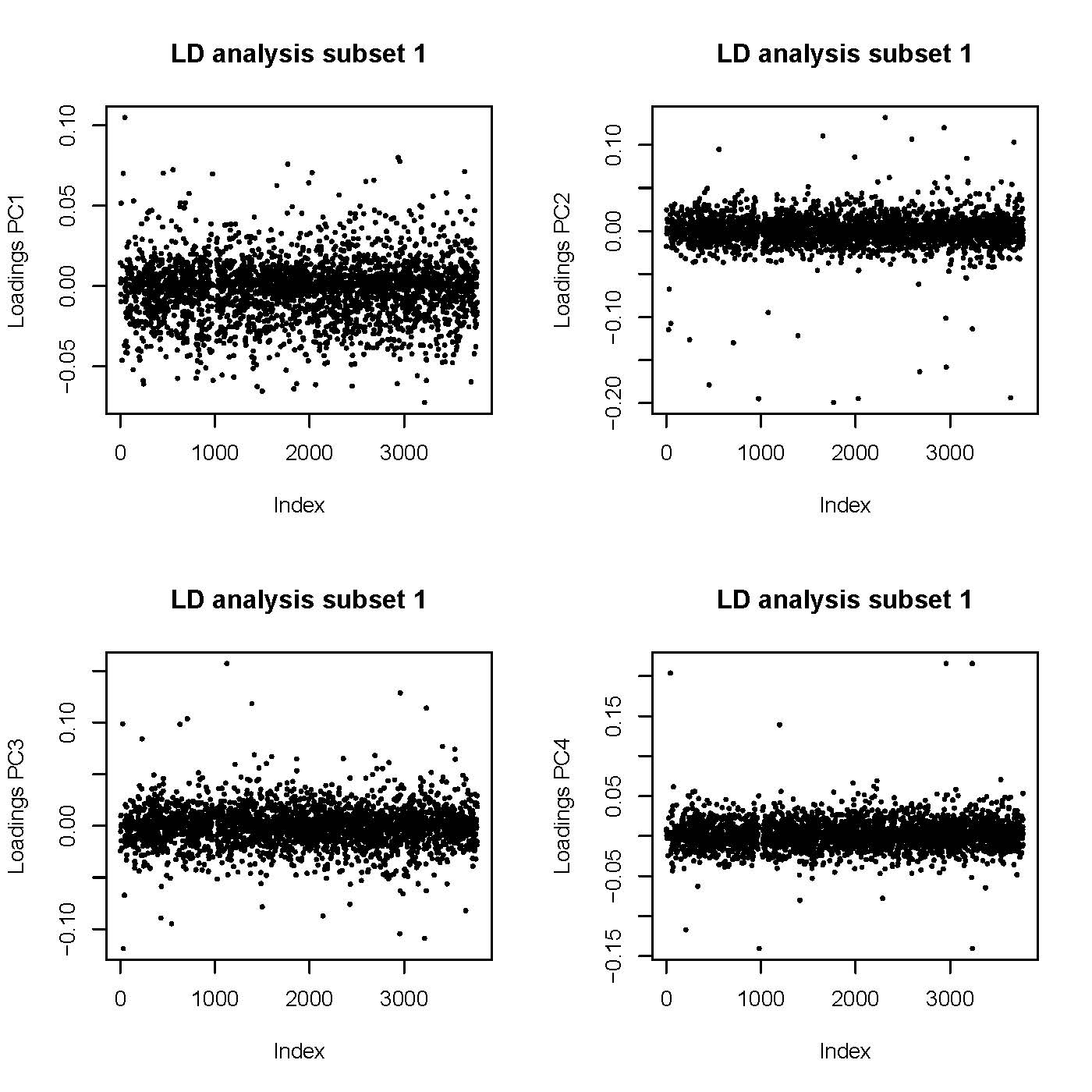

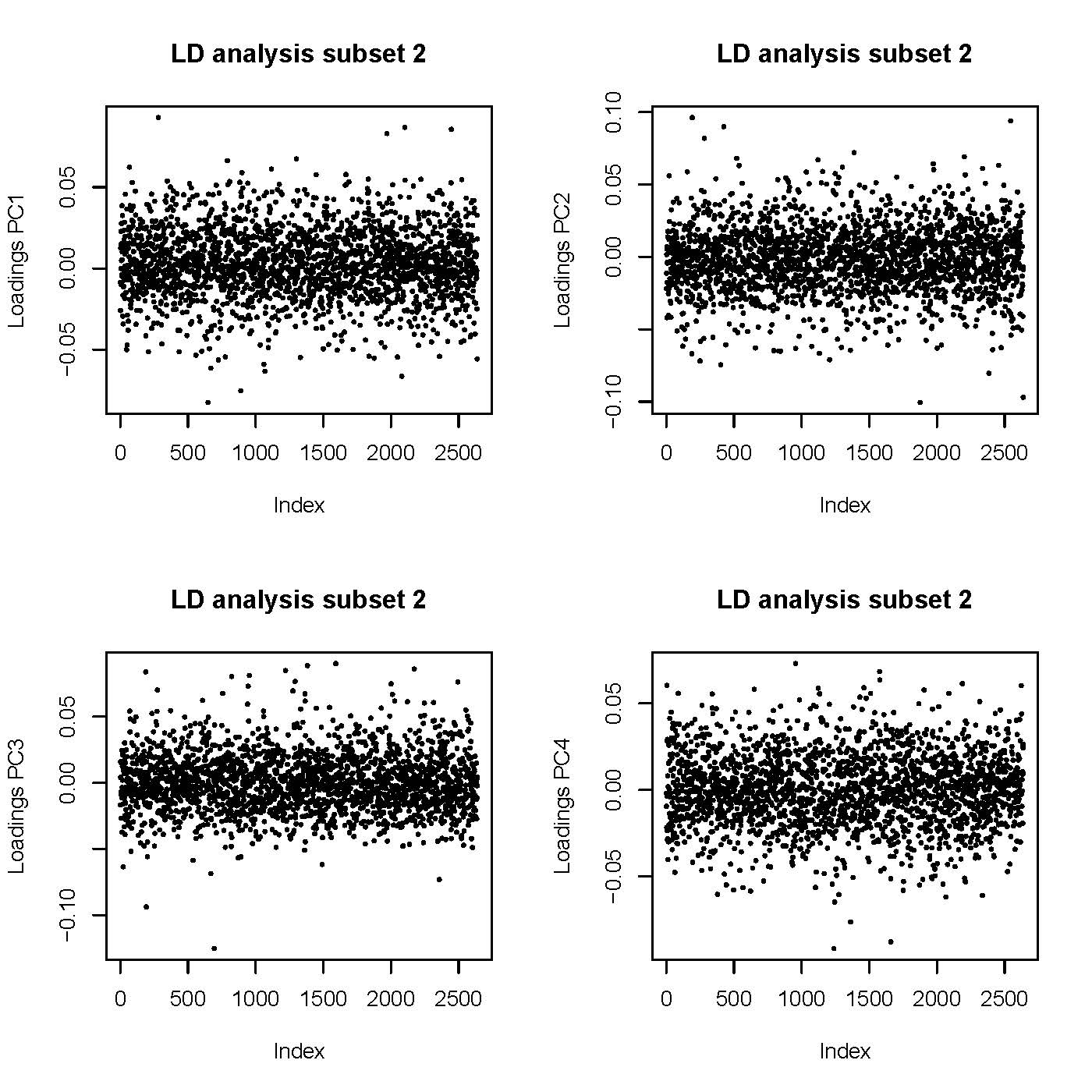

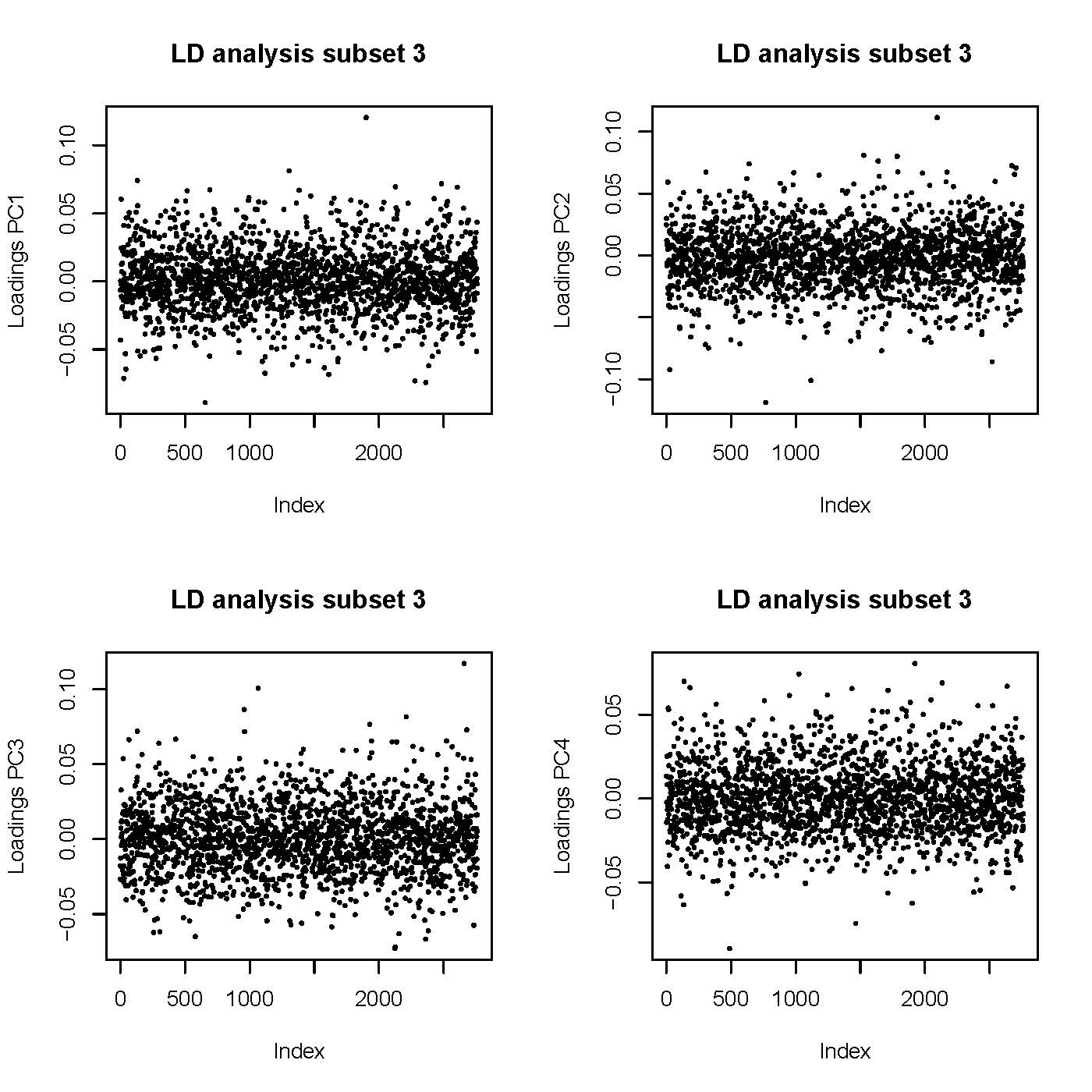

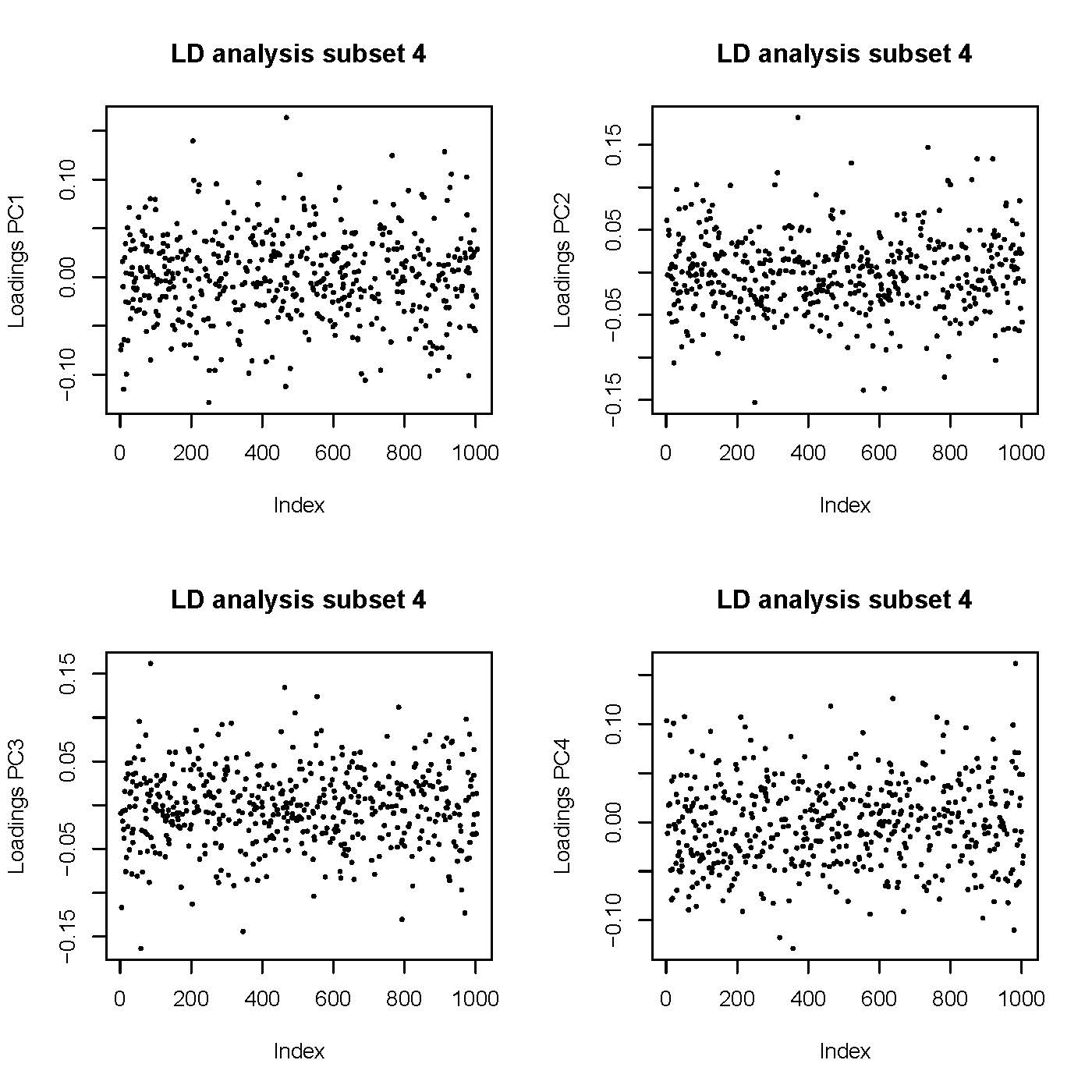

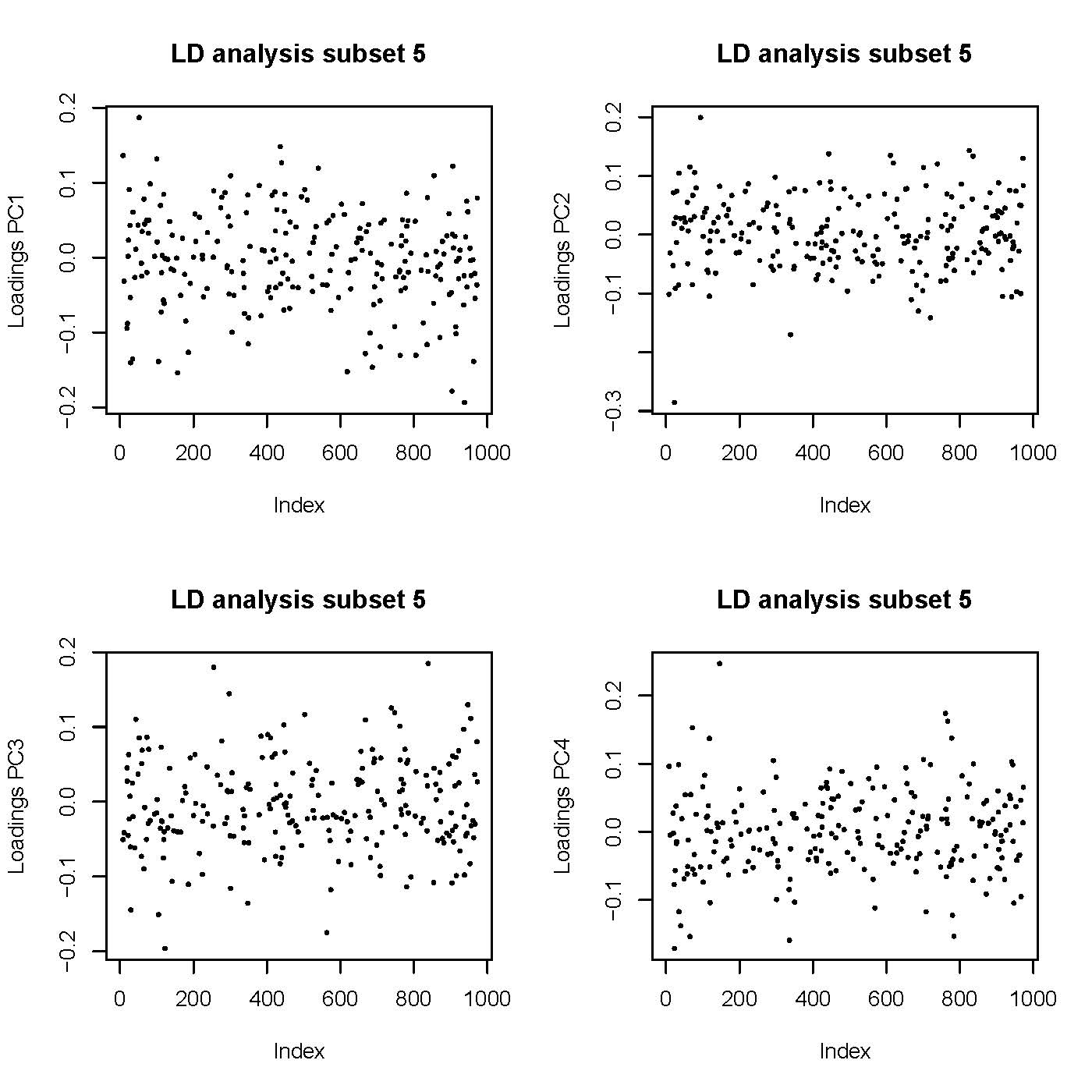


# Appendix S2: Environmental association drivers

The methodological process to determine the functional spatial scales for environmental association analyses described in chapter 2.4 (Fig. S5; Stephane Dray et al., 2021) resulted in 5 significant moran eigenvector maps (Table S4). However, MEM3 was correlated to the max. temperature of the warmest month (Bio 5) and MEM2 was correlated to the precipitation seasonality (Bio 15; Table S4). Therefore, the correlated MEMs were excluded from further analyses. The remaining variables (Fig. S6; Fig S7) used in the RDA and gradient forest analyses were not correlated (r<0.6; Table S4).


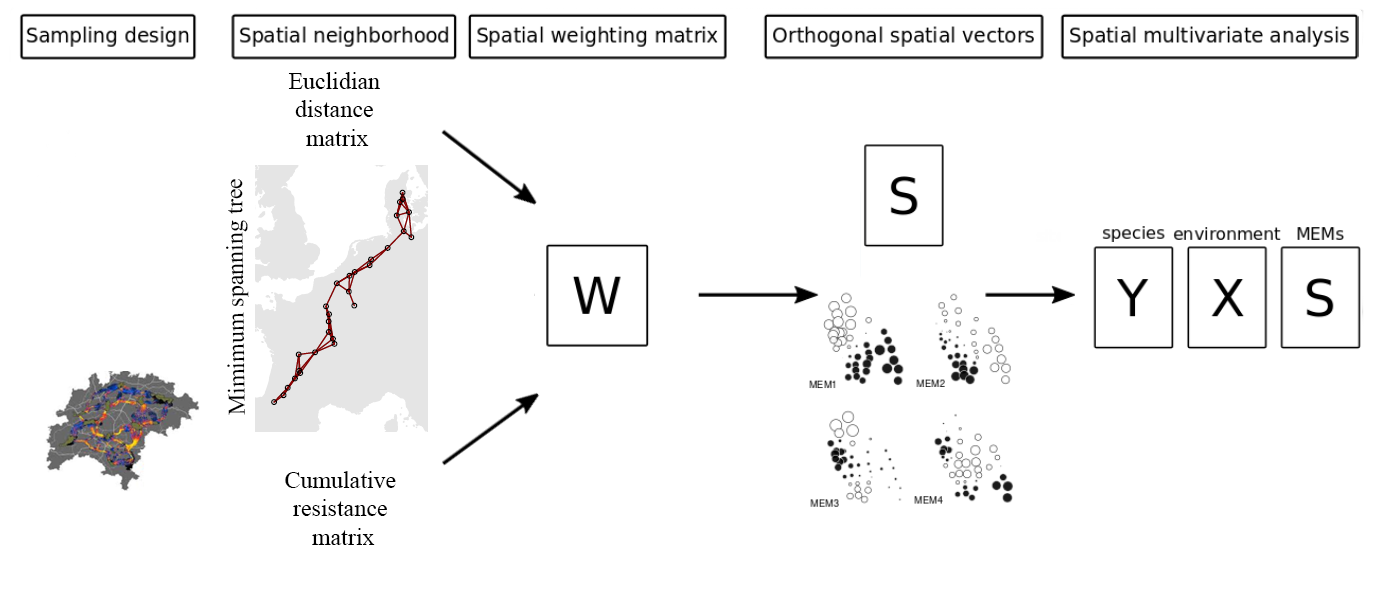
Figure S5: Method diagram for quantifying Moran's eigenvector maps based on the cumulative resistance matrix and the Euclidian distance matrix (based on Dray et al., 2021).

Table S4: Spearman correlation coefficients of climate and spatial variables.

|  |  | Bio_05 | Bio_15 | MEM3 | MEM4 | MEM1 | MEM5 | MEM2 |
| --- | --- | --- | --- | --- | --- | --- | --- | --- |
| Bio_05 |  | 1 | -0.34 | -0.73 | 0.04 | -0.45 | -0.02 | -0.31 |
| Bio_15 |  | -0.34 | 1 | -0.16 | -0.31 | 0.36 | -0.21 | 0.68 |
| MEM3 |  | -0.73 | -0.16 | 1 | 0 | 0 | 0 | 0 |
| MEM4 |  | 0.04 | -0.31 | 0 | 1 | 0 | 0 | 0 |
| MEM1 |  | -0.45 | 0.36 | 0 | 0 | 1 | 0 | 0 |
| MEM5 |  | -0.02 | -0.21 | 0 | 0 | 0 | 1 | 0 |
| MEM2 |  | -0.31 | 0.68 | 0 | 0 | 0 | 0 | 1 |

Figure S6: Eigenvalues of the selected spatial Moran eigenvector maps and the plot scores in geographical space.


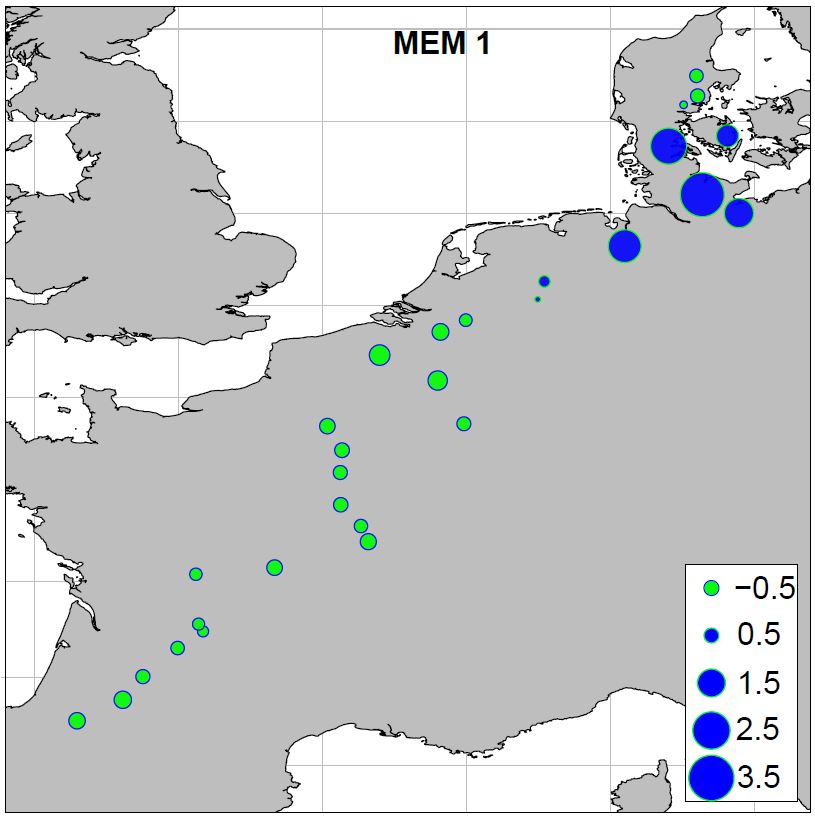

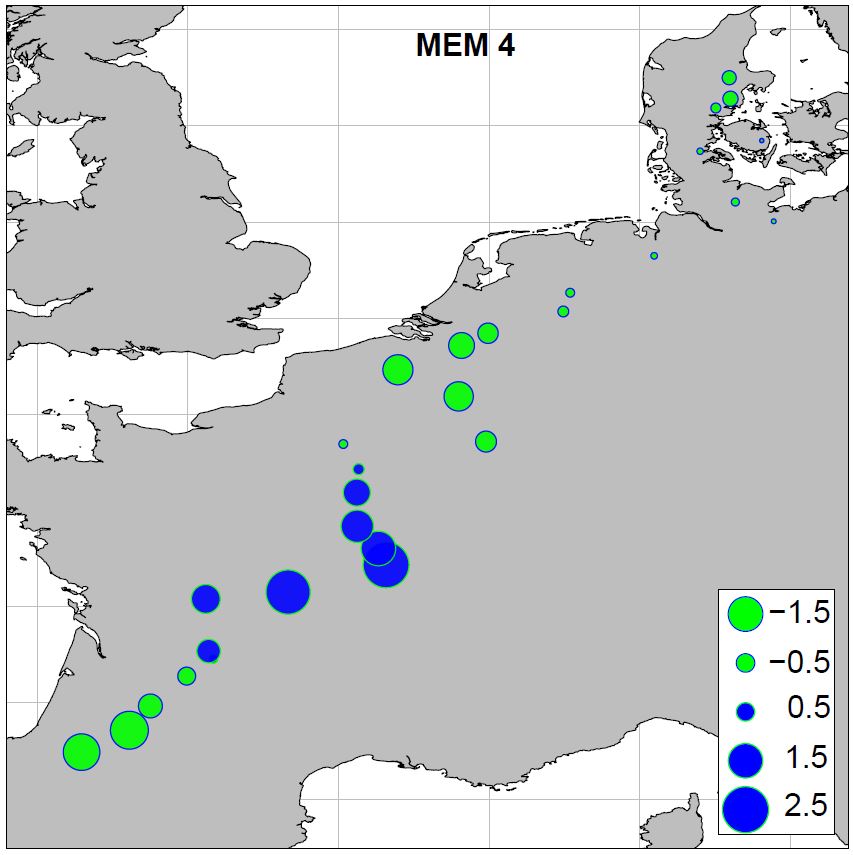

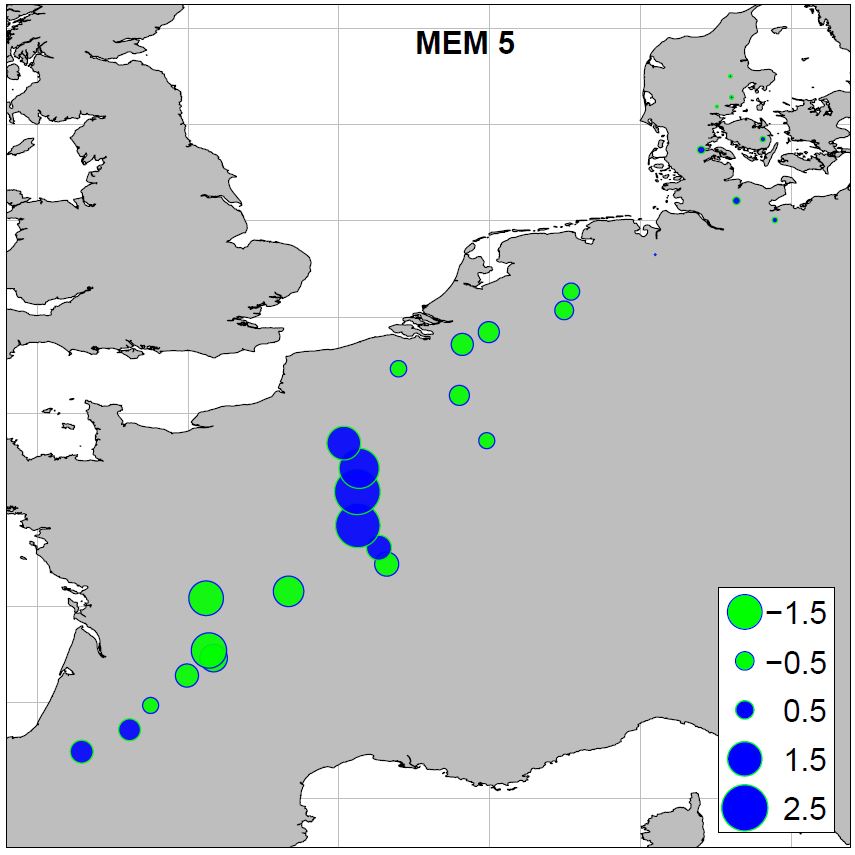

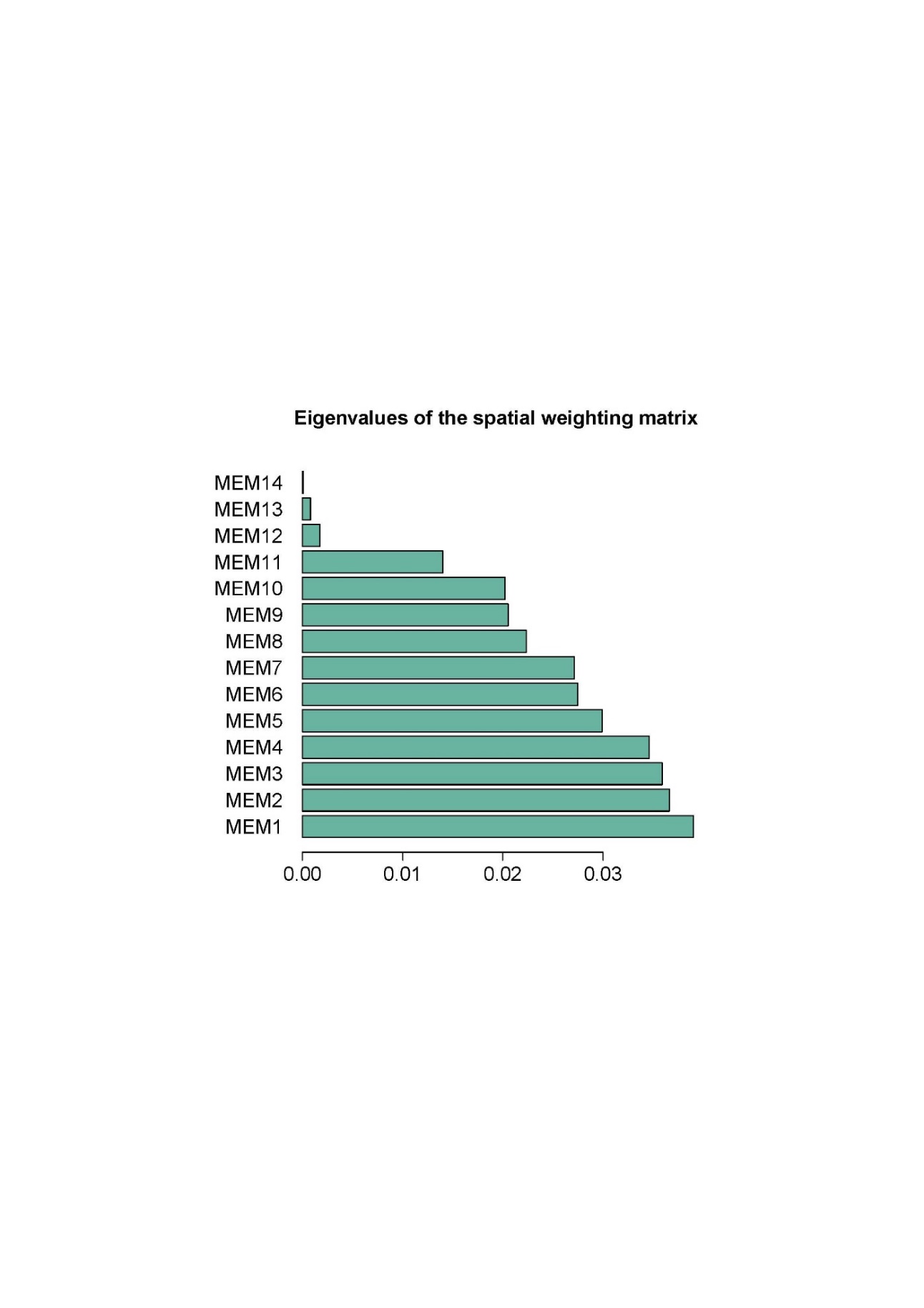


Figure S7: Distribution of plots and their relation to the maximum temperature of the warmest month (Bio 5) and precipitation seasonality (Bio 15).


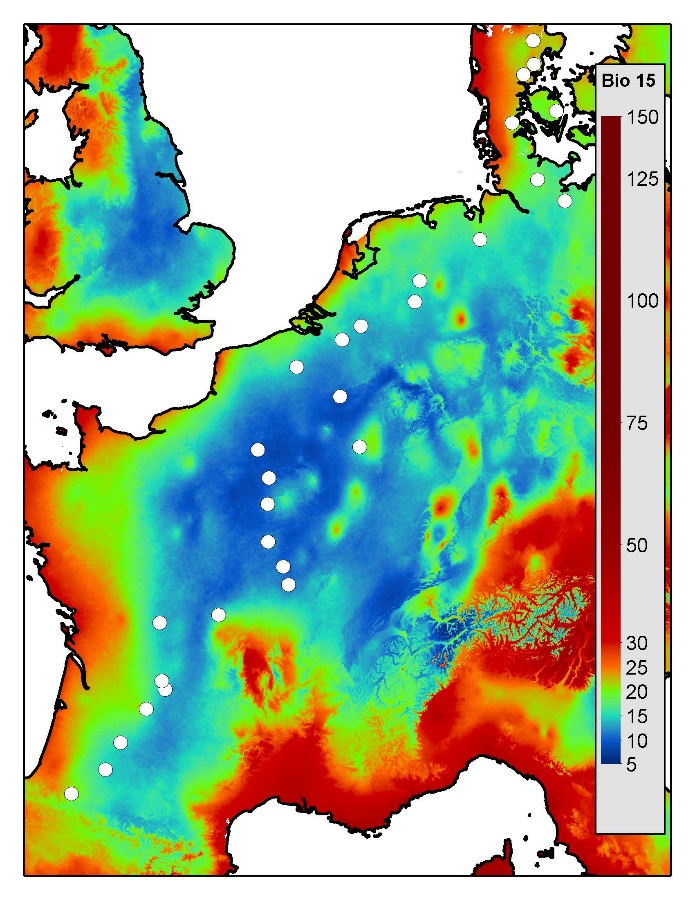

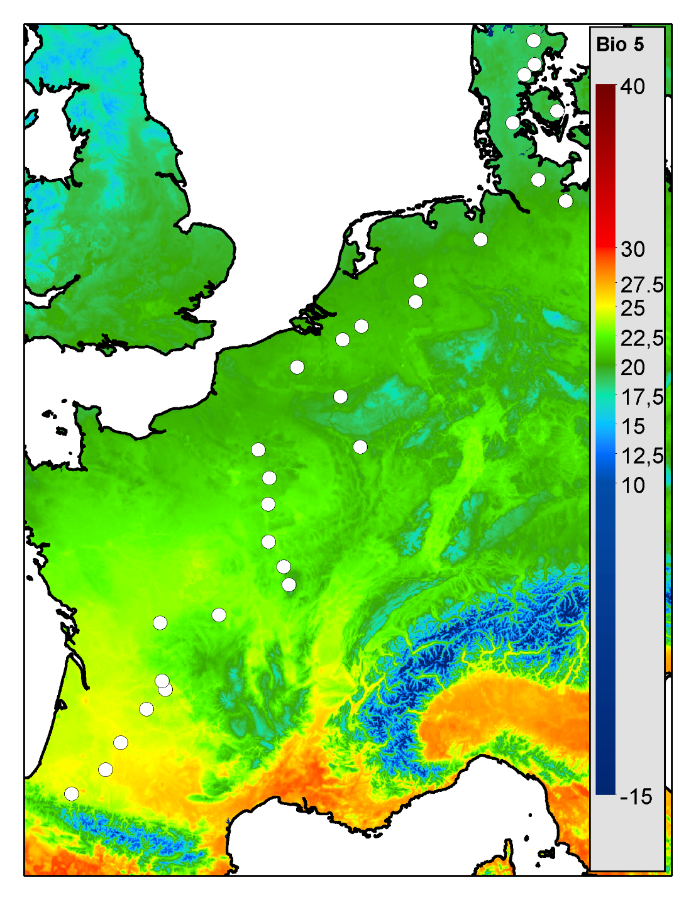


# Appendix S3: Indications for post-glacial (re)colonization

The population structure analysis (sNMF) indicated major clustering with sharp genetic turnover between southern regions and a large degree of mixture between central and north European populations. These results (Fig. 2) imply post-glacial (re)colonization of northern Europe from multiple southern refugia (Hewitt, 1999; Sommer & Zachos, 2009), which is substantiated by the PCAdapt axes score visualisation (Fig. S8; Luu, Bazin, & Blum, 2017). The genetic turnover of southern populations, characterized by PC1, is most likely determined by a long demographic history and local adaptation. The genetic variation determined by the post-glacial (re)colonization is depicted by PC1 scores and illustrated a very low genetic differentiation, even though there is a high amount of dispersal limitation in northern landscapes (Van Daele, Honnay, & De Kort, 2021). Furthermore, the theory of post-glacial (re)colonization of *P. elatior* is also reflected in the northwards decrease (Fig. S9) of the average genetic distance (between all sampled populations). This could also explain why the adaptive genetic diversity is lower in the south than in the north of the distribution range (Fig. 5).


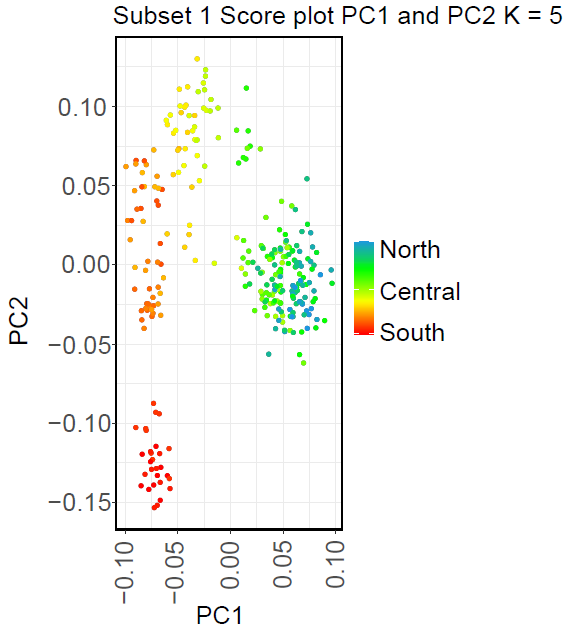


Figure S8: Biplot of PCAdapt on the first subset with 5 retained principal components(K = 5).


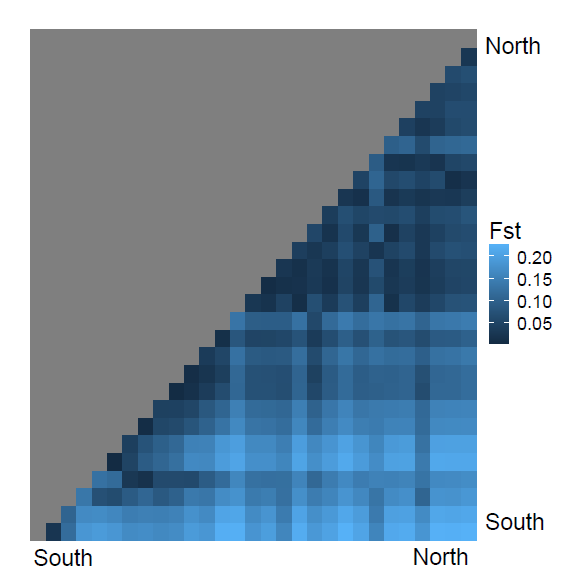

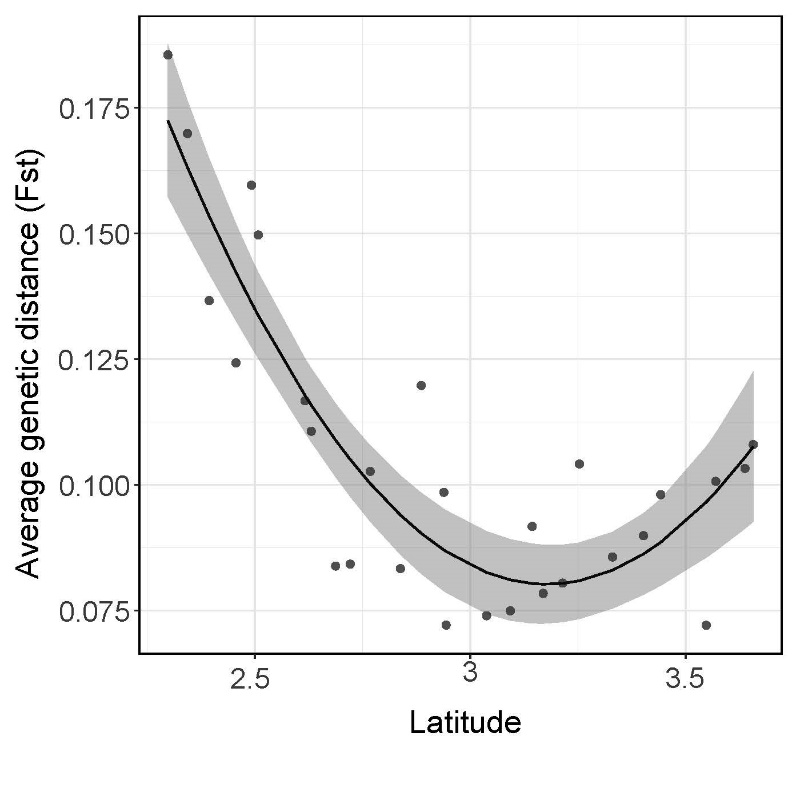


Figure S9: The relation between the average genetic distance (between all populations)

# Appendix S4: SNP and GO ontology outliers

Table S5: Overview of climate outliers, as determined by max temperature of the warmest month and the precipitation seasonality in the partial RDA analysis. The SNP name is a concatenation of the contig name (contig_xxx) and the SNP position on the contig. The SNP subset determines on which SNP subset the data analyses were executed. The library effect is a categorical factor where 0 indicates that no library effects were detected and an outlier SNP was thus detected in all of the increasingly restrictive datasets. When the categorical factor indicates 1 then the outlier SNP was not detected or retained in the most restrictive dataset, where all SNPs with at least one sequencing error were removed. When the categorical factor indicates 2 then the outlier SNP was only detected or retained in the main dataset. The library effects in the main text were determined by the proportion of each category in the complete dataset. The uncertainty of the missing data indicates the error rate (percentage) of the imputation strategy. The false discovery rate of the distinct analyses are also indicated in percentages.

| SNP name | SNP subset | Library effect | Uncertainty missing data | FDR Bayescan | FDR PCAdapt | FDR pRDA climate |
| --- | --- | --- | --- | --- | --- | --- |
| contig_22614_105 | 1 | 1 | 0.0 | 0.1 | 16.2 | 0.1 |
| contig_62_3411 | 1 | 0 | 0.0 | 0.1 | 5.7 | 0.1 |
| contig_7337_580 | 1 | 0 | 0.0 | 0.1 | 0.1 | 0.1 |
| contig_119392_704 | 1 | 0 | 0.8 | 0.1 | 14.4 | 0.2 |
| contig_2685_522 | 1 | 0 | 0.0 | 0.1 | 0.1 | 0.2 |
| contig_40512_3299 | 1 | 1 | 0.0 | 0.1 | 0.2 | 0.2 |
| contig_76131_120 | 1 | 1 | 0.0 | 0.1 | 25.8 | 0.2 |
| contig_4972_460 | 1 | 0 | 0.0 | 0.1 | 1.6 | 0.5 |
| contig_17268_124 | 1 | 1 | 0.0 | 0.1 | 14.2 | 0.6 |
| contig_21441_1023 | 2 | 0 | 5.9 | 0.1 | 0.2 | 1 |
| contig_5533_1888 | 2 | 0 | 5.1 | 0.1 | 49.2 | 1 |
| contig_8105_545 | 2 | 0 | 0.6 | 0.1 | 0.1 | 1 |
| contig_13303_217 | 1 | 0 | 0.1 | 1.5 | 100 | 1.1 |
| contig_16315_442 | 1 | 1 | 0.0 | 0.1 | 0.5 | 1.1 |
| contig_1937_9770 | 1 | 1 | 0.0 | 0.1 | 0.1 | 1.1 |
| contig_2646_162 | 1 | 0 | 0.0 | 0.1 | 9.8 | 1.1 |
| contig_2646_498 | 1 | 0 | 0.0 | 0.1 | 0.1 | 1.1 |
| contig_63389_332 | 1 | 1 | 0.1 | 0.1 | 0.5 | 1.1 |
| contig_83902_9 | 1 | 0 | 0.0 | 0.1 | 1.3 | 1.1 |
| contig_4814_71 | 1 | 0 | 2.7 | 100 | 100 | 1.2 |
| contig_6222_584 | 1 | 1 | 6.3 | 0.1 | 100 | 1.2 |
| contig_29872_138 | 2 | 0 | 5.0 | 0.1 | 0.5 | 1.6 |
| contig_12342_637 | 2 | 0 | 1.8 | 0.1 | 0.1 | 1.7 |
| contig_110457_1553 | 1 | 0 | 0.0 | 0.1 | 0.1 | 1.8 |
| contig_15803_38 | 1 | 0 | 2.2 | 0.2 | 100 | 1.8 |
| contig_33978_182 | 1 | 0 | 3.5 | 0.2 | 100 | 1.8 |
| contig_79719_615 | 1 | 1 | 0.9 | 0.8 | 100 | 1.8 |
| contig_4056_70 | 1 | 0 | 0.0 | 0.1 | 0.3 | 2.3 |
| contig_7337_837 | 1 | 0 | 0.0 | 0.1 | 0.1 | 2.3 |
| contig_37498_594 | 1 | 2 | 2.3 | 0.1 | 100 | 2.4 |
| contig_42968_829 | 1 | 1 | 0.0 | 0.1 | 100 | 2.8 |
| contig_5475_204 | 1 | 1 | 0.0 | 0.1 | 0.1 | 2.8 |
| contig_8204_654 | 1 | 0 | 0.0 | 0.1 | 100 | 2.8 |
| contig_2685_153 | 1 | 0 | 0.0 | 0.1 | 28.8 | 2.9 |
| contig_60913_119 | 1 | 1 | 0.0 | 100 | 100 | 2.9 |
| contig_17745_4707 | 1 | 0 | 1.4 | 0.1 | 6.8 | 3 |
| contig_935_2120 | 1 | 1 | 0.0 | 0.1 | 0.1 | 3 |
| contig_5649_365 | 4 | 2 | 6.2 | 100 | 7.8 | 3.1 |
| contig_184_450 | 1 | 0 | 2.3 | 0.1 | 100 | 3.7 |
| contig_57411_188 | 1 | 1 | 2.4 | 0.1 | 30.5 | 3.7 |
| contig_32825_864 | 1 | 2 | 1.4 | 0.1 | 12.4 | 4.2 |
| contig_1540_368 | 1 | 0 | 0.0 | 0.1 | 3.7 | 4.3 |
| contig_2646_260 | 1 | 0 | 0.0 | 0.1 | 23.1 | 4.6 |
| contig_4973_871 | 1 | 1 | 0.0 | 0.1 | 32.5 | 4.6 |
| contig_14835_13638 | 1 | 1 | 4.7 | 0.6 | 100 | 4.7 |
| contig_35752_230 | 1 | 0 | 1.4 | 0.1 | 0.1 | 4.7 |
| contig_413_861 | 1 | 0 | 2.9 | 0.1 | 100 | 4.7 |
| contig_7371_479 | 1 | 0 | 0.0 | 0.1 | 0.1 | 4.7 |
| contig_209_639 | 1 | 0 | 1.8 | 1.6 | 100 | 4.9 |
| contig_2646_376 | 1 | 0 | 0.0 | 0.1 | 30.9 | 4.9 |
| contig_29019_1082 | 1 | 0 | 1.3 | 0.5 | 100 | 4.9 |
| contig_6097_143 | 1 | 1 | 0.0 | 3.7 | 29.1 | 4.9 |
| contig_68986_366 | 1 | 1 | 2.9 | 4 | 16.9 | 4.9 |

Table S6: Significant biological processes as determined by the max. temperature of the warmest month and the precipitation seasonality in the partial RDA (pRDA). Statistical evaluation of significant go terms (KS classic, KS elim, Fisher classic and Fisher parent/child) depict the p values and significant p values (< 0.05) are indicated in light green shading and dark green text. Significant GO terms that were only detected by the GO analysis of pRDA outliers are indicated in orange. Significant GO terms that were detected by the GO analysis of pRDA outliers and also detected by Bayescan are indicated in yellow. Significant GO terms that were detected by the GO analysis of pRDA outliers and also detected by PCAdapt are indicated in blue. Significant GO terms that were detected by all three of the outlier analyses are indicated in green.

| GO.ID | Term | Annotated | Significant | Expected | KS classic | KS elim | Fisher classic | Fisher par/child |
| --- | --- | --- | --- | --- | --- | --- | --- | --- |
| GO:0006725 | cellular aromatic compound metabolic process | 47 | 2 | 1.11 | **0.026** | **0.007** | 0.623 | 0.253 |
| GO:1901360 | organic cyclic compound metabolic process | 47 | 2 | 1.11 | **0.026** | **0.007** | 0.623 | 0.187 |
| GO:0006139 | nucleobase-containing compound metabolic process | 46 | 2 | 1.08 | **0.040** | **0.011** | 0.609 | 0.193 |
| GO:0046483 | heterocycle metabolic process | 46 | 2 | 1.08 | **0.040** | **0.011** | 0.609 | 0.242 |
| GO:1901135 | carbohydrate derivative metabolic process | 5 | 1 | 0.12 | **0.049** | **0.049** | 0.192 | 0.091 |
| GO:0015074 | DNA integration | 24 | 0 | 0.56 | 0.053 | **0.018** | 1.000 | 1.000 |
| GO:0006259 | DNA metabolic process | 25 | 0 | 0.59 | 0.074 | **0.032** | 1.000 | 1.000 |
| GO:0008152 | metabolic process | 122 | 2 | 2.87 | 0.231 | **0.008** | 0.998 | 0.986 |
| GO:0008150 | biological_process | 146 | 4 | 3.44 | 0.359 | **0.000** | 0.992 | 1.000 |
| GO:0044281 | small molecule metabolic process | 8 | 2 | 0.19 | 0.055 | 0.086 | **0.036** | **0.004** |
| GO:0006812 | cation transport | 6 | 2 | 0.14 | 0.303 | 0.399 | **0.020** | 0.714 |
| GO:0006811 | ion transport | 7 | 2 | 0.16 | 0.367 | 0.472 | **0.028** | 0.318 |
| GO:1901566 | organonitrogen compound biosynthetic process | 15 | 2 | 0.35 | 0.427 | 0.531 | 0.118 | **0.046** |
| GO:0051179 | localization | 12 | 2 | 0.28 | 0.526 | 0.367 | 0.079 | **0.034** |


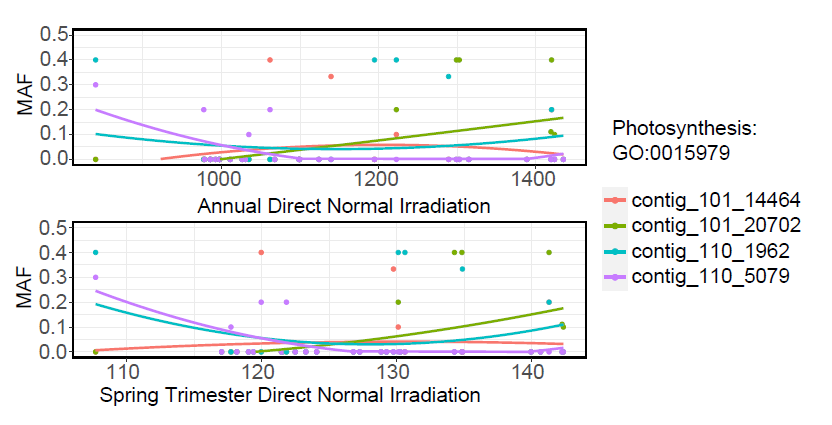


Figure S10: Relation between irradiance and photosynthesis SPN frequencies (Copernicus Atmosphere Monitoring Service, 2020).

# References

Beaumont, M. A., & Balding, D. J. (2004). Identifying adaptive genetic divergence among populations from genome scans. *Molecular Ecology*, *13*(4), 969–980. doi: 10.1111/j.1365-294X.2004.02125.x

Copernicus Atmosphere Monitoring Service. (2020). Monthly and yearly average Direct Normal Irradiation maps from CAMS Radiation Service over Europe. Retrieved from AGATE website: http://www.soda-pro.com/help/cams-services/cams-radiation-service/download-europe-volume#agate-maps

Dray, S., Bauman, D., Blanchet, G., Borcard, D., Clappe, S., Guenard, G., … Wagner, H. H. (2021). *Adespatial: Multivariate Multiscale Spatial Analysis*. Retrieved from https://cran.r-project.org/package=adespatial

Hewitt, G. M. (1999). Post-glacial re-colonization of European biota. *Biological Journal of the Linnean Society*, *68*(1–2), 87–112. doi: 10.1006/bijl.1999.0332

Luu, K., Bazin, E., & Blum, M. G. B. (2017). pcadapt: an R package to perform genome scans for selection based on principal component analysis. *Molecular Ecology Resources*, *17*(1), 67–77. doi: 10.1111/1755-0998.12592

Mastretta-Yanes, A., Arrigo, N., Alvarez, N., Jorgensen, T. H., Piñero, D., & Emerson, B. C. (2015). Restriction site-associated DNA sequencing, genotyping error estimation and de novo assembly optimization for population genetic inference. *Molecular Ecology Resources*, *15*(1), 28–41. doi: 10.1111/1755-0998.12291

O’Leary, S. J., Puritz, J. B., Willis, S. C., Hollenbeck, C. M., & Portnoy, D. S. (2018). These aren’t the loci you’e looking for: Principles of effective SNP filtering for molecular ecologists. *Molecular Ecology*, *27*(16), 3193–3206. doi: 10.1111/mec.14792

Sommer, R. S., & Zachos, F. E. (2009). Fossil evidence and phylogeography of temperate species: “Glacial refugia” and post-glacial recolonization. *Journal of Biogeography*, *36*(11), 2013–2020. doi: 10.1111/j.1365-2699.2009.02187.x

Van Daele, F., Honnay, O., & De Kort, H. (2021). The role of dispersal limitation and reforestation in shaping the distributional shift of a forest herb under climate change. *Diversity and Distributions*, *27*(9), 1775–1791. doi: 10.1111/ddi.13367
